# Supplementary material for: Heme-binding enables allosteric modulation in an ancient TIM-barrel glycosidase
Source: Nat Commun. 2021 Jan 15;12:380. doi: 10.1038/s41467-020-20630-1 (PMC7810902; doi:10.1038/s41467-020-20630-1)
Supplement: Supplementary file 1 — Supplementary Information [file 41467_2020_20630_MOESM1_ESM.pdf]

# SUPPLEMENTARY INFORMATION FOR

## Heme-binding enables allosteric modulation in an ancient TIM-barrel glycosidase

*Gloria Gamiz-Arco[1,7], Luis I. Gutierrez-Rus[1,7], Valeria A. Risso[1], Beatriz Ibarra-Molero[1], Yosuke Hoshino[2], Dušan Petrović[3,8], Jose Justicia[4], Juan Manuel Cuerva[4], Adrian Romero-Rivera[3], Burckhard Seelig[5], Jose A. Gavira[6], Shina C.L. Kamerlin[3]*, Eric A. Gaucher[2]*, Jose M. Sanchez-Ruiz[1]**

[1]Departamento de Quimica Fisica. Facultad de Ciencias, Unidad de Excelencia de Quimica Aplicada a Biomedicina y Medioambiente (UEQ), Universidad de Granada, 18071 Granada, Spain.

[2]Department of Biology, Georgia State University, Atlanta, GA 30306 U.S.A.

[3]Science for Life Laboratory, Department of Chemistry-BMC, Uppsala University, BMC Box 576, S-751 23 Uppsala, Sweden.

[4]Departamento de Quimica Organica. Facultad de Ciencias, Unidad de Excelencia de Quimica Aplicada a Biomedicina y Medioambiente (UEQ), Universidad de Granada, 18071 Granada, Spain.

[5]Department of Biochemistry, Molecular Biology, and Biophysics, University of Minnesota, Minneapolis, Minnesota, United States of America, & BioTechnology Institute, University of Minnesota, St. Paul, Minnesota, United States of America.

[6]Laboratorio de Estudios Cristalograficos, Instituto Andaluz de Ciencias de la Tierra, CSIC, Unidad de Excelencia de Quimica Aplicada a Biomedicina y Medioambiente (UEQ), Universidad de Granada, Avenida de las Palmeras 4, Granada 18100 Armilla, Spain.

[7]These authors contributed equally to this work

[8]Current address: Hit Discovery, Discovery Sciences, Biopharmaceutical R&D, AstraZeneca, 431 50 Gothenburg, Sweden.

*email: lynn.kamerlin@kemi.uu.se or egaucher@gsu.edu or sanchezr@ugr.es

## Supplementary Methods

### Homology model preparation

For the reconstructed ancestral sequence for node 72, 3D structures were initially prepared using homology modelling. In searching for the suitable templates, several criteria were imposed, i.e., high sequence identity[1,2] (>= 50%) and query sequence coverage (>= 90%), as determined by BLAST and HHBlits[1,2], as well as the oligomerization state of a monomer and belonging to the GH1 family in the CAZY database. The following PDB structures fulfilled the criteria, and were used to create homology models with SWISS-MODEL[3-5]: family 1 β-glucosidase from *Thermotoga maritima* (1W3J), β-glucosidase A from *Clostridium cellulovorans* (3AHX), engineered β-glucosidase from soil metagenome (3CMJ), GH1 β-glucosidase Td2F2 (3WH5), and β-glucosidase 1A from *Thermotoga neapolitana* (5IDI). The quality of the five homology models was assessed using the Swiss-Model provided scores and the structural parameters estimated with MolProbity[6] and the models from 1W3J, 3CMJ, and 5IDI templates were selected for the further work. While the protein core (the $(\beta\alpha)_8$ barrel) was generally modelled very well, major differences could be observed between the homology models in the region of the catalytic loops.

## Supplementary Tables

**Table S1.** Sequence identity of the ancestral glycosidase with the modern glycosidases in the set used as starting point for ancestral sequence reconstruction. The sequence identity values with the ancestral protein span the 0.26-0.59 range.

| | |
|---|---|
| Ancestral | 1,000 |
| Euk-XP_002676353.1.Naegleria.gruberi.NEG-M.Oth | 0,483 |
| Euk-OLQ05614.1.Symbiodinium.microadriaticum.SAR | 0,463 |
| Euk-OCF22674.1.Kwoniella.bestiolae.CBS.10118.Fg | 0,439 |
| Euk-KXS17974.1.Gonapodya.prolifera.JEL478.Fg.435 | 0,366 |
| Euk-KOO32978.1.Chrysochromulina.CCMP291.Oth.458 | 0,431 |
| Euk-KNC76787.1.Sphaeroforma.arctica.JP610.Met.475 | 0,389 |
| Euk-GAX29444.1.Fistulifera.solaris.SAR.446 | 0,519 |
| Euk-GAX16517.1.Fistulifera.solaris.SAR.450 | 0,500 |
| Euk-ETP15771.1.Phytophthora.parasitica.CJ01A1.SAR | 0,364 |
| Euk-ETP11111.1.Phytophthora.parasitica.CJ01A1.SAR | 0,516 |
| Euk-ETP02644.1.Phytophthora.parasitica.CJ01A1.SAR | 0,366 |
| Euk-EOD40853.1.Emiliania.huxleyi.CCMP1516.Oth.401 | 0,249 |
| Euk-EOD13795.1.Emiliania.huxleyi.CCMP1516.Oth.457 | 0,432 |
| Euk-EIE19776.1.Coccomyxa.subellipsoidea.C-169.Arpl | 0,271 |
| Euk-CHR-CAC34952.1.Piromyces.E2.Fg.463 | 0,393 |
| Euk-CHR-CAC08178.1.Homo.sapiens.Met.452 | 0,419 |
| Euk-CHR-BAO04178.1.Delphinium.grandiflorum.Arpl | 0,418 |
| Euk-CHR-BAE87009.1.Phanerochaete.chrysosporium.Fg | 0,464 |
| Euk-CHR-BAE63197.1.Aspergillus.oryzae.RIB40.Fg.460 | 0,440 |
| Euk-CHR-AGS32242.1.Coptotermes.gestroi.Met.460 | 0,424 |
| Euk-CHR-AAP13852.1.Bombyx.mori.Met.462 | 0,398 |
| Euk-CHR-AAL92115.1.Glycine.max.Arpl.456 | 0,377 |
| Euk-CHR-AAL37719.1.Solanum.lycopersicum.Arpl.461 | 0,435 |
| Euk-CHR-AAL25999.1.Brevicoryne.brassicae.Met.453 | 0,419 |
| Euk-CHR-AAK62412.1.Arabidopsis.thaliana.Arpl.465 | 0,404 |
| Euk-CHR-AAK32833.1.Arabidopsis.thaliana.Arpl.462 | 0,392 |
| Euk-CBJ30694.1.Ectocarpus.siliculosus.SAR.450 | 0,489 |
| Euk-AAG46031.1.Leishmania.infantum.Exc.456 | 0,371 |
| CPR-OYX53756.1.Sacchari.32-50-13.Oth.420 | 0,303 |
| CPR-OHA99561.1.Zambryski.R-O2_12_FULL_43_12b.Pc | 0,345 |
| CPR-OHA32072.1.Taylor.R-O2_01_FULL_45_15b.Pc | 0,340 |
| CPR-OHA13338.1.Taga.R-O2_01_FULL_39_11.Pc.405 | 0,317 |
| CPR-OHA09246.1.Sung.R-O2_01_FULL_59_16.Pc.399 | 0,311 |
| CPR-OGZ46118.1.Ryan.R-O2_01_FULL_48_27.Pc.416 | 0,330 |
| CPR-OGZ07021.1.Lloyd.R-O2_02_FULL_50_13.Pc.385 | 0,328 |

| | |
|---|---|
| CPR-OGZ06701.1.Lloyd.R-O2_02_FULL_50_11.Pc.387 | 0,332 |
| CPR-OGZ02593.1.Lipton.R-O2_01_FULL_53_13.Pc.396 | 0,315 |
| CPR-OGY99744.1.Lipton.R-O2_01_FULL_52_25.Pc.404 | 0,320 |
| CPR-OGY99381.1.Lipton.R-O2_01_FULL_52_25.Pc.410 | 0,305 |
| CPR-OGY71081.1.Jackson.R-O2_01_FULL_44_13.Pc.395 | 0,300 |
| CPR-OGY40781.1.Brenner.RIFOXYD1_FULL_41_16.Pc.389 | 0,312 |
| CPR-OGM99997.1.Yanofsky.R-O2_01_FULL_41_53.Pc.416 | 0,322 |
| CPR-OGM98994.1.Yanofsky.R-O2_01_FULL_41_26.Pc.386 | 0,314 |
| CPR-OGM90535.1.Wolfe.R-O2_01_FULL_38_11.Pc.412 | 0,336 |
| CPR-OGM00912.1.Uhr.RIFOXYC2_FULL_47_19.Pc.406 | 0,334 |
| CPR-OGL39085.1.Sacchari.R-O2_02_FULL_46_7.Oth | 0,311 |
| CPR-OGL30033.1.Sacchari.R-O2_02_FULL_47_12.Oth | 0,308 |
| CPR-OGK55604.1.Roizman.R-O2_01_FULL_45_11.Mc | 0,325 |
| CPR-OGJ70814.1.Peri.R-O2_12_FULL_53_10.Oth.390 | 0,318 |
| CPR-OGG92310.1.Kuenen.R-O2_12_FULL_42_13.Pc.406 | 0,316 |
| CPR-OGG87996.1.Kaiser.RIFOXYD1_FULL_42_15.Pc.382 | 0,312 |
| CPR-OGG55752.1.Kaiser.R-O2_01_FULL_55_37.Pc.387 | 0,317 |
| CPR-OGG06799.1.Gottesman.R-O2_01_FULL_42_12.Mc.399 | 0,326 |
| CPR-OGF90942.1.Giovannoni.R-O2_02_FULL_45_14.Pc | 0,351 |
| CPR-OGE98567.1.Doudna.R-O2_12_FULL_42_9.Pc.394 | 0,348 |
| CPR-OGE83632.1.Doudna.R-O2_02_FULL_43_13b.Pc.393 | 0,322 |
| CPR-OGE24950.1.Davies.R-O2_02_FULL_39_12.Mc.398 | 0,316 |
| CPR-OGE15085.1.Davies.GWA1_38_6.Mc.403 | 0,336 |
| CPR-KUK76511.1.WS6.34_10.Oth.396 | 0,338 |
| CPR-KKS47395.1.Giovannoni.GW2011_GWF2_42_19.Pc.397 | 0,351 |
| CPR-KKS25793.1.Jorgensen.GW2011_GWF2_41_8.Pc.408 | 0,332 |
| CPR-KKQ84990.1.Woese.GW2011_GWB1_38_8.Mc.399 | 0,347 |
| CPR-KKQ36955.1.Woese.GW2011_GWA1_37_7.Mc.396 | 0,353 |
| CPR-KKQ29214.1.Nomura.GW2011_GWA1_37_20.Pc.379 | 0,365 |
| CPR-KKQ12362.1.Moran.GW2011_GWF1_36_78.Pc.403 | 0,333 |
| Bac-WP_081838630.1.Thermogem.carboxidivorans.Clf | 0,511 |
| Bac-WP_053225736.1.Solirubrobacter.soli.Ac.440 | 0,483 |
| Bac-WP_033274371.1.Actinospica.acidiphila.Ac.393 | 0,313 |
| Bac-WP_028863797.1.Psychromonas.aquimarina.gP.448 | 0,415 |
| Bac-WP_026735568.1.Fischerella.PCC.9605.Cy.448 | 0,467 |
| Bac-WP_026389483.1.Acholeplasma.multilocale.Tn.438 | 0,336 |
| Bac-WP_016873414.1.Chlorogloeopsis.fritschii.Cy | 0,470 |
| Bac-WP_010471747.1.Acaryochloris.CCMEE.5410.Cy.441 | 0,488 |
| Bac-SEK57728.1.Rhodococcus.maanshanensis.Ac.387 | 0,300 |
| Bac-OUQ12786.1.Massiliomicrobiota.An142.Fm.454 | 0,449 |
| Bac-OUQ09514.1.Massiliomicrobiota.An142.Fm.466 | 0,340 |
| Bac-OIO58018.1.CG1_02_48_14.Mn.415 | 0,349 |

| | |
|---|---|
| Bac-OGS21660.1.RIFOXYA2_FULL_39_19.El.449 | 0,500 |
| Bac-OGF51021.1.RIFOXYA2_FULL_40_8.Frs.449 | 0,457 |
| Bac-OAA30738.1.Kosmotoga.arenicorallina.S304.Tg | 0,300 |
| Bac-KPM53585.1.Frankia.R43.Ac.388 | 0,322 |
| Bac-KIX85561.1.JCVI.TM6SC1.Dp.402 | 0,294 |
| Bac-GAT31492.1.Terrimicrobium.sacchariphilum.V.449 | 0,503 |
| Bac-EGF89970.1.Asticcacaulis.biprosthecum.C19.aP | 0,293 |
| Bac-EFH82146.1.Ktedonobacter.racemifer.Clf | 0,503 |
| Bac-CRX38655.1.Estrella.lausannensis.Chl.421 | 0,269 |
| Bac-CRH90323.1.Chlamydia.trachomatis.Chl.449 | 0,401 |
| Bac-CHR-CAC47470.1.Sinorhizobium.meliloti.1021.aP | 0,483 |
| Bac-CHR-BAC96154.1.Vibrio.vulnificus.YJ016.gP.445 | 0,481 |
| Bac-CHR-BAB37196.1.Escherichia.coli.Sakai.gP.464 | 0,366 |
| Bac-CHR-BAB36995.1.Escherichia.coli.Sakai.gP.462 | 0,346 |
| Bac-CHR-AIY95585.1.Bacillus.subtilis.Fm | 0,353 |
| Bac-CHR-AIY95329.1.Bacillus.subtilis.Fm | 0,365 |
| Bac-CHR-AIY91871.1.Bacillus.subtilis.Fm | 0,437 |
| Bac-CHR-AIY91623.1.Bacillus.subtilis.Fm | 0,473 |
| Bac-CHR-AGA60135.1.Microbacterium.Gsoil167.Ac.398 | 0,288 |
| Bac-CHR-AEI42200.1.Paenibacillus.mucilaginosus.Fm | 0,296 |
| Bac-CHR-ADD27066.1.Meiothermus.ruber.DSM.1279.DT | 0,524 |
| Bac-CHR-ADD01617.1.Thermoanaerobacter.italicus.Fm | 0,453 |
| Bac-CHR-ACV58907.1.Alicyclobaci.acidocaldarius.Fm | 0,551 |
| Bac-CHR-ACO44852.1.Deinococcus.deserti.VCD115.DT | 0,497 |
| Bac-CHR-ACM06095.1.Thermomicrobium.roseum.Clf | 0,521 |
| Bac-CHR-ACL70277.1.Halothermothrix.orenii.H.168.Fm | 0,557 |
| Bac-CHR-ACI19973.1.Dictyoglomus.thermophilum.Dg | 0,581 |
| Bac-CHR-ABU56651.1.Roseiflexus.castenholzii.Clf | 0,541 |
| Bac-CHR-ABR73190.1.Marinomonas.MWYL1.gP.444 | 0,449 |
| Bac-CHR-ABJ60960.1.Lactobacillus.gasseri.Fm | 0,340 |
| Bac-CHR-ABJ59900.1.Lactobacillus.gasseri.Fm | 0,494 |
| Bac-CHR-ABJ59596.1.Lactobacillus.gasseri.Fm | 0,362 |
| Bac-CHR-ABD82858.1.Saccharophagus.degradans.gP | 0,497 |
| Bac-CHR-ABD80656.1.Saccharophagus.degradans.gP | 0,443 |
| Bac-CHR-AAN58797.1.Streptococcus.mutans.UA159.Fm | 0,346 |
| Bac-CHR-AAK78365.1.Clostridium.acetobutylicum.Fm | 0,509 |
| Bac-CHR-AAG59862.1.Sphingomonas.paucimobilis.aP | 0,261 |
| Bac-CHR-AAB49339.1.Fusobacterium.mortiferum.Fs.442 | 0,525 |
| Bac-CHR-AAA24815.1.Dickeya.chrysanthemi.gP.456 | 0,365 |
| Bac-CCB88561.1.Simkania.negevensis.Z.Chl.422 | 0,270 |
| Bac-CAB95278.1.Streptomyces.coelicolor.A3_2.Ac.444 | 0,526 |
| Bac-BAY28878.1.Nostoc.carneum.NIES-2107.Cy.452 | 0,471 |

| | |
|---|---|
| Bac-BAM04503.1.Phycisphaera.mikurensis.Pl | 0,464 |
| Bac-BAL98072.1.Caldilinea.aerophila.DSM.14535.Clf | 0,522 |
| Bac-APF18099.1.Caldithrix.abyssi.DSM.13497.Cld.414 | 0,313 |
| Bac-AFG36462.1.Spirochaeta.africana.DSM.8902.Sp | 0,331 |
| Bac-AEP25088.1.Thermotoga.maritima.MSB8.Tg.443 | 0,593 |
| Bac-ADD01635.1.Thermoanaerobacter.italicus.Ab9.Fm | 0,578 |
| Bac-ADB52696.1.Conexibacter.woesei.DSM.14684.Ac | 0,496 |
| Bac-ACZ10042.1.Sebaldella.termitidis.ATCC.33386.Fs | 0,390 |
| Bac-ACZ09962.1.Sebaldella.termitidis.ATCC.33386.Fs | 0,399 |
| Bac-ACL69240.1.Halothermothrix.orenii.H.168.Fm.417 | 0,339 |
| Bac-ACI21065.1.Thermodesulfovib.yellowstonii.Nsr | 0,336 |
| Bac-ABJ83756.1.Solibacter.usitatus.Ellin6076.Ad | 0,305 |
| Bac-ABG04991.1.Rubrobacter.xylanophilus.Ac | 0,524 |
| Bac-AAK79373.1.Clostridium.acetobutylicum.Fm | 0,359 |
| Arc-SMD30897.1.Picrophilus.oshimae.Ery-Thp | 0,256 |
| Arc-Seed-BAA29440.1.Pyrococcus.horikoshii.Ery-Thc | 0,377 |
| Arc-OYT35865.1.Archaeoglobales.ex4484_92.Ery-Ach | 0,334 |
| Arc-OWP55065.1.Cuniculiplasma.C_DKE.Ery-Thp.470 | 0,263 |
| Arc-KYH41278.1.Bathyarchaeota.B26-2.Ery-Unc.477 | 0,278 |
| Arc-KJE49247.1.Acidiplasma.MBA-1.Ery-Thp.464 | 0,237 |
| Arc-CHR-ADL19795.1.Acidilo.saccharovorans.TK-Thp | 0,311 |
| Arc-CHR-ABW01492.1.Caldivir.maquilingensis.TK-Thp | 0,295 |
| Arc-CHR-AAL81332.1.Pyrococcus.furiosus.Ery-Thc | 0,294 |
| Arc-CHR-AAL80566.1.Pyrococcus.furiosus.Ery-Thc | 0,392 |
| Arc-CHR-AAL80197.1.Pyrococcus.furiosus.Ery-Thc | 0,275 |
| Arc-CHR-AAK43121.1.Sulfolobus.solfataricus.TK-Thp | 0,290 |
| Arc-CHR-AAD43138.1.Thermosphaera.aggregans.TK-Thp | 0,272 |
| Arc-BAB59827.1.Thermoplasma.volcanium.GSS1.Ery-Thp | 0,265 |
| Arc-AJB42198.1.Thermofilum.carboxyditrophus.TK-Thp | 0,285 |
| Arc-AJB41496.1.Thermofilum.carboxyditrophus.TK-Thp | 0,277 |
| Arc-AIF16120.1.marine.thaumarchaeote.TK-Thm | 0,345 |

**Table S2**. Catalytic parameters for the hydrolysis of 4-nitrophenyl-β-D-glucopyranoside and 4-nitrophenyl-β-D-galactopyranoside at pH 7 (HEPES buffer 100 mM) and 25 ºC catalyzed by modern and ancestral family 1 glycosidases. For each enzyme/substrate combination, the independent experimental replicates (involving at least two enzyme preparations) were performed. Values of catalytic parameters derived from the fitting of the Michaelis-Menten equation (See Figures S6-S9) are given for each replicate. Errors are standard deviations derived from the fits. The average values of the three replicates are given in Table S3.

| | 4-nitrophenyl-β-D-glucopyranoside | | | 4-nitrophenyl-β-D-galactopyranoside | | |
|---|---|---|---|---|---|---|
| | $k_{cat}$ ($s^{-1}$) | $K_M$ (mM) | $k_{cat}/K_M$ ($s^{-1}$ $mM^{-1}$) | $k_{cat}$ ($s^{-1}$) | $K_M$ (mM) | $k_{cat}/K_M$ ($s^{-1}$ $mM^{-1}$) |
| Ancestral glycosidase | 0.1739 ± 0.0074 | 6.190 ± 0.673 | 0.0281 ± 0.0020 | 0.1159 ± 0.0066 | 33.61 ± 3.75 | 0.00345 ± 0.00020 |
| | 0.1543 ± 0.0054 | 5.701 ± 0.580 | 0.0271 ± 0.0019 | 0.1233 ± 0.0087 | 31.74 ± 4.48 | 0.00388 ± 0.00029 |
| | 0.1578 ± 0.0071 | 4.76 ± 0.58 | 0.0331 ± 0.0028 | 0.1215 ± 0.0045 | 32.76 ± 2.39 | 0.00371 ± 0.00014 |
| Ancestral with bound heme | 0.521 ± 0.022 | 17.18 ± 1.31 | 0.0303 ± 0.0011 | 0.2801 ± 0.0056 | 21.57 ± 1.03 | 0.01298 ± 0.00037 |
| | 0.443 ± 0.034 | 16.18 ± 2.25 | 0.0274 ± 0.0018 | 0.2269 ± 0.0065 | 12.55 ± 1.15 | 0.0181 ± 0.0012 |
| | 0.414 ± 0.032 | 18.65 ± 2.40 | 0.0222 ± 0.0012 | 0.2321 ± 0.0074 | 16.89 ± 1.42 | 0.01374 ± 0.00075 |
| *Halothermothrix orenii* | 17.21 ± 0.25 | 0.3248 ± 0.022 | 52.99 ± 3.11 | 94.8 ± 4.3 | 26.14 ± 2.59 | 3.63 ± 0.20 |
| | 16.41 ± 0.42 | 0.342 ± 0.039 | 48.03 ± 4.71 | 62.54 ± 2.67 | 14.45 ± 1.85 | 4.33 ± 0.38 |
| | 13.285 ± 0.587 | 0.399 ± 0.071 | 33.33 ± 4.83 | 64.59 ± 1.25 | 14.40 ± 0.78 | 4.49 ± 0.16 |
| | 18.44 ± 0.27 | 0.367 ± 0.025 | 50.18 ± 2.84 | 25.99 ± 0.75 | 9.70 ± 0.94 | 2.68 ± 0.19 |

| | | | | | | |
|---|---|---|---|---|---|---|
| *Thermotoga maritima* | 17.92 ± 0.16 | 0.348± 0.015 | 51.55 ± 1.82 | 30.77 ± 1.66 | 15.44 ± 2.41 | 1.99 ± 0.21 |
| | 17.77 ± 0.24 | 0.366 ± 0.022 | 48.48 ± 2.45 | 28.41 ± 0.73 | 10.36 ± 0.87 | 2.74 ± 0.17 |
| *Marinomonas sp.* (strain MWYL1) | 10.54 ± 0.22 | 0.095 ± 0.015 | 110.15 ± 16.20 | 28.51 ± 0.80 | 14.68 ± 1.14 | 1.94 ± 0.10 |
| | 10.38 ± 0.20 | 0.089 ± 0.013 | 116.43 ± 15.10 | 22.26 ± 0.70 | 13.20 ± 1.21 | 1.69 ± 0.11 |
| | 10.62 ± 0.41 | 0.112± 0.030 | 94.47 ± 22.80 | 24.96 ± 0.57 | 12.19 ± 0.89 | 2.05 ± 0.11 |
| *Saccharophagus degradans* (strain 2-40$^T$) | 25.19 ± 0.34 | 0.710 ± 0.053 | 35.46 ± 2.34 | 5.87 ± 0.29 | 22.20 ± 2.53 | 0.264 ± 0.018 |
| | 20.55 ± 0.30 | 0.545 ± 0.042 | 37.71 ± 2.58 | 7.21 ± 0.62 | 32.87 ± 5.46 | 0.219 ± 0.019 |
| | 21.09 ± 0.52 | 0.549 ± 0.070 | 38.43 ± 4.33 | 6.02 ± 0.20 | 22.20 ± 1.68 | 0.271 ± 0.012 |

**Table S3.** Catalytic parameters for the hydrolysis of 4-nitrophenyl-β-D-glucopyranoside and 4-nitrophenyl-β-D-galactopyranoside at pH 7 (HEPES buffer 100 mM) and 25 ºC catalyzed by modern and ancestral family 1 glycosidases. The values shown here are the average values, together with the corresponding standard deviations, of three independent replicates (see Table S2). n = 3 independent determinations of the Michaelis-Menten profiles.

| | 4-nitrophenyl β-D-glucopyranoside | | | 4-nitrophenyl β-D-galactopyranoside | | |
|---|---|---|---|---|---|---|
| | $k_{cat}$ ($s^{-1}$) | $K_M$ (mM) | $k_{cat}/K_M$ ($s^{-1}$ $mM^{-1}$) | $k_{cat}$ ($s^{-1}$) | $K_M$ (mM) | $k_{cat}/K_M$ ($s^{-1}$ $mM^{-1}$) |
| Ancestral | 0.1620 ± 0.0085 | 5.55 ± 0.59 | 0.0294 ± 0.0026 | 0.1202 ± 0.0032 | 32.70 ± 0.76 | 0.00368 ± 0.00018 |
| Ancestral with bound heme | 0.459 ± 0.045 | 17.3 ± 1.0 | 0.0266 ± 0.0036 | 0.246 ±0.024 | 17.0 ± 3.7 | 0.0149 ± 0.0023 |
| *Halothermothrix orenii* | 15.6 ± 1.7 | 0.355 ± 0.032 | 44.8 ± 8.4 | 74 ± 15 | 18.3 ± 5.5 | 4.15 ± 0.37 |
| *Thermotoga maritima* | 18.04 ± 0.29 | 0.3603 ± 0.0087 | 50.1 ± 1.3 | 28.4 ± 2.0 | 11.8 ± 2.6 | 2.47 ± 0.34 |
| *Marinomonas sp.* (strain MWYL1) | 10.51 ± 0.10 | 0.0980 ± 0.0097 | 107.0 ± 9.2 | 25.2 ± 2.6 | 13.4 ± 1.0 | 1.89 ± 0.15 |
| *Saccharophagus degradans* (strain 2-40) | 22.3 ± 2.1 | 0.601 ± 0.077 | 37.2 ± 1.3 | 6.37 ± 0.60 | 25.8 ± 5.0 | 0.251 ± 0.023 |

**Table S4.** Amino acid residues at critical active site positions in modern and ancestral glycosidases. The catalytic carboxylic acids, as well as the positions involved in the binding of the glycone and aglycone moieties of the substrate[7] are shown. Residues at those positions in the ancestral glycosidase and the modern glycosidase from *Halothermothrix orenii* are given. The last column provides the residue statistics for the set of modern glycosidases used as starting point for ancestral sequence reconstruction. Glycosidases are known to be somewhat specific for the glycone moiety of the substrate and much less specific for the aglycone moiety, which is reflected in a lower residue conservation at the protein residues involved in aglycone binding. See Figure S10 for a graphical illustration.

| | Ancestral glycosidase | Modern glycosidase from *Halothermothrix orenii* | Sequence statistics in the set of modern glycosidases used as a starting point for ancestral sequence reconstruction. The residue present in the ancestral protein is highlighted in bold |
|---|---|---|---|
| Catalytic residues | E171 | E166 | **E 98%** Q 0.7% R 0.7% S 0.7% |
| | E358 | E354 | **E 100%** |
| Residues involved in binding the glycone part of the substrate | Q25 | Q20 | **Q 98.7%** H 0.7% P 0.7% |
| | H126 | H121 | **H 97.3%** W 1.3% D 0.7% N 0.7% |
| | N170 | N165 | **N 99.3%** C 0.7% |
| | W404 | W401 | **W 98.7%** M 0.7% R 0.7% |
| | E411 | E408 | **E 84.7%** S 14.7% R 0.7% |
| | W412 | W409 | **W 86.7%** A 5.3% F 2.7% L 1.3% T 1.3% I 0.7% M 0.7% S 0.7% V 0.7% |
| Residues involved in binding the aglycone part of the substrate | W40 | W35 | **W 85.3%** S 2.7% V 2.7% A 2.0% I 2.0% F 1.3% L 1.3% M 1.3% C 0.7% T 0.7% |
| | F43 | F38 | **F 36%** W 36% Y 6.7% L 5.3% A 4.7% M 2.7% E 1.3% G 1.3% K 1.3% Q 1.3% V 1.3% I 0.7% S 0.7% T 0.7% |
| | W127 | W122 | **W 50.7%** F 39.3% Y 7.3% G 0.7% H 0.7% L 0.7% S 0.7% |
| | F175 | V170 | Y 21.3% V 18.0% **F 17.3%** S 12.0% P 6.0% I 4.7% A 4.0% M 3.3% L 2.7% Q 2.7% T 2.7% Q 2.7% T 2.7% W 2.7% N 2% C 0.7% |
| | L178 | E173 | **L 30.7%** M 10.0% G 8.0% N 8.0% A 6.7% Q 6.7% H 4.7% F 4.0% E 3.3% C 2.7% K 2.7% S 2.7% Y 2.0% D 1.3% P 1.3% R 1.3% T 1.3% V 1.3% W 0.7% |
| | H185 | H180 | **H 26.7%** W 21.3% F 20.7% L 5.3% Y 4.0% K 3.3% M 2.7% S 2.7% G 2.0% I 2.0% Q 2.0% R 2.0% A 1.3% D 1.3% V 1.3% E 0.7% N 0.7% |
| | N227 | N222 | **N 48.7%** A 16.0% H 8.0% S 6.0% D 4.7% L 3.3% I 2.0% Q 2.0% T 2.0% V 2.0% F 1.3% Y 1.3% C 0.7% E 0.7% G 0.7% R 0.7% |

| | | | |
|---|---|---|---|
| | W332 | W327 | **W 86%** Y 6.7% E 1.3% L 1.3% F 0.7% G 0.7% H 0.7% I 0.7% R 0.7% S 0.7% V 0.7% |
| | A413 | A410 | **A 40.7%** S 8.0% E 6.7% L 6.7% T 6.7% D 5.3% G 5.3% H 4.0% I 3.3% N 3.3% R 2.0% V 2.0% F 1.3% P 1.3% Q 1.3% C 0.7% K 0.7% M 0.7% |
| | F420 | F417 | **F 80.0%** Y 18.0% L 1.3% R 0.7% |

**Table S5.** Estimated rates of the hydrolysis of several substrates catalysed by the ancestral glycosidase at node 72. All substrates were assayed at concentration of 0.1 mM, 25 ºC, HEPES buffer 50 mM pH 7 with a concentration of enzyme of 4-5 μM. Rates were calculated from the time dependence of the absorbance due to the released 4-nitrophenolate after correction for the blank. A value of ~0 is reported when no significant rate enhancement over the blank was detected.

| **Substrate** | **Rate $s^{-1}$** |
|---|---|
| *4*-nitrophenyl-β-D-glucopyranoside | 1.1E-02 |
| *4*-nitrophenyl-β-D-galactopyranoside | 2.4E-03 |
| *4*-nitrophenyl-β-D-fucopyranoside | 3.4E-02 |
| *4*-nitrophenyl-β-D-mannopyranoside | 1.7E-04 |
| *4*-nitrophenyl-β-D-xylopyranoside | 7.9E-05 |
| *4*-nitrophenyl-β-D-glucuronide | 1.0E-04 |
| *4*-nitrophenyl-β-D-ribofuranoside | 3.1E-04 |
| *4*-nitrophenyl-β-D-thioglucopyranoside | ~0 |
| *4*-nitrophenyl-β-D-cellobioside | 1.3E-04 |
| *4*-nitrophenyl-β-D-lactopyranoside | 5.2E-05 |
| *4*-nitrophenyl-β-D-maltoside | 4.7E-05 |
| *4*-nitrophenyl-β-D-galactopyranoside 6P | ~0 |
| *4*-nitrophenyl-N-acetil-β-D-glucosamine | ~0 |
| *4*-nitrophenyl-N-acetil-β-D galactosamine | ~0 |
| *4*-nitrophenyl-α-D-maltoside | ~0 |
| *4*-nitrophenyl-α-L-fucopyranoside | ~0 |
| *4*-nitrophenyl-α-D-maltohexaoside | ~0 |
| *4*-nitrophenyl-α-D-galactopyranoside | ~0 |
| *4*-nitrophenyl-α-D-glucopyranoside | ~0 |
| *4*-nitrophenyl-α-D-xylopyranoside | ~0 |
| *4*-nitrophenyl-α-D-mannopyranoside | ~0 |
| *4*-nitrophenyl-α-D-rhamnopyranoside | ~0 |

**Table S6.** Statistics of amino acid occurrence in modern family 1 glycosidases at the positions involved in interactions with the heme in the ancestral glycosidase. The set of sequences used as starting point for ancestral reconstruction has been used for this calculation. The number of occurrences for the predicted ancestral residues are highlighted in bold. Note that, in all cases, the ancestral residue is the most common residue (i.e., the consensus residue) in the modern set. Still, in all positions other amino acid residues are also observed.

| | Ala | Arg | Asn | Asp | Cys | Gln | Glu | Gly | His | Ile | Leu | Lys | Met | Phe | Pro | Ser | Thr | Trp | Tyr | Val |
|---|---|---|---|---|---|---|---|---|---|---|---|---|---|---|---|---|---|---|---|---|
| **N173** | 5 | 2 | **42** | 4 | - | 2 | 8 | 8 | - | 7 | 7 | 1 | 13 | 6 | - | 4 | 7 | 29 | 4 | 1 |
| **A199** | **37** | 7 | 6 | - | - | 5 | 2 | 14 | - | 8 | 15 | 4 | 10 | 7 | - | 10 | 7 | - | 14 | 4 |
| **L202** | 6 | - | - | - | - | 13 | 5 | - | - | 22 | **62** | 1 | 16 | 4 | - | 2 | 7 | - | - | 12 |
| **L203** | 22 | 1 | 4 | - | - | 1 | - | - | 1 | 32 | **62** | - | 7 | 10 | - | 2 | - | - | 1 | 7 |
| **H206** | - | - | 2 | - | - | 2 | - | - | **132** | 1 | - | - | - | - | - | 13 | - | - | - | - |
| **I224** | 4 | - | 3 | - | 9 | - | - | 1 | - | **72** | 18 | - | 6 | 6 | 5 | 4 | 2 | 5 | - | 15 |
| **L226** | 1 | - | 11 | 2 | - | 1 | 3 | - | 19 | 16 | **46** | 21 | 3 | 7 | 6 | 2 | - | - | 10 | 2 |
| **L228** | 9 | - | 17 | 1 | 6 | 1 | - | 13 | 1 | 13 | **27** | 1 | 11 | 15 | 2 | 8 | 4 | 1 | 7 | 13 |
| **F251** | 3 | 10 | 5 | 4 | - | 2 | 3 | - | 2 | 12 | 21 | 1 | 2 | **55** | - | 1 | 3 | 7 | 10 | 7 |
| **N252** | 6 | 10 | **11** | 3 | - | 11 | 8 | 5 | 11 | 3 | 5 | 4 | 2 | 17 | - | 11 | 9 | 13 | 5 | 4 |
| **F255** | 2 | - | - | - | 2 | - | - | - | 1 | 10 | 3 | - | 1 | **91** | - | 1 | - | 8 | 14 | 5 |
| **L256** | 13 | 1 | - | 2 | - | - | 1 | 7 | - | 16 | **57** | - | 8 | 11 | 4 | 5 | 4 | 2 | 4 | 4 |
| **K261** | 2 | 20 | 6 | 2 | 2 | - | 3 | 3 | - | 1 | 5 | **23** | - | 6 | - | 4 | 11 | 1 | 5 | - |
| **Y264** | - | - | - | - | - | - | - | - | - | 4 | 7 | 1 | 1 | 9 | - | 1 | - | - | **75** | 1 |
| **L296** | 7 | - | 8 | 6 | 5 | 12 | - | 2 | 8 | 16 | **32** | - | 2 | 6 | 4 | 12 | 6 | - | 8 | 16 |
| **S295** | 3 | 7 | 1 | 6 | 3 | 7 | 2 | 7 | 6 | 2 | 3 | 20 | - | 1 | 26 | **22** | 20 | - | 14 | - |
| **R345** | 6 | **26** | 6 | 16 | - | 5 | 20 | 3 | 7 | 2 | 4 | 11 | 3 | 5 | - | 1 | 4 | 15 | 14 | 2 |
| **Y350** | 8 | 4 | 7 | 3 | 2 | 3 | - | 11 | 3 | - | 8 | 3 | 1 | 10 | 4 | 4 | 6 | - | **63** | 1 |

**Table S7**. Statistics of number of amino acid differences between modern family 1 glycosidases and the ancestral glycosidases at the positions involved in heme binding in the latter. The set of sequences used as starting point for ancestral reconstruction has been used for this calculation (see Table S6). Note that all the modern sequences differ from the ancestral sequence in a significant number of positions.

| number of identical amino acids | **1** | **2** | **3** | **4** | **5** | **6** | **7** | **8** | **9** | **10** | **11** | **12** | **13** | **14** | **15** | **16** | **17** | **18** |
|---|---|---|---|---|---|---|---|---|---|---|---|---|---|---|---|---|---|---|
| sequences with identical amino acids | - | 2 | 17 | 22 | 26 | 14 | 25 | 18 | 13 | 4 | 6 | 3 | - | - | - | - | - | - |

**Table S8.** Atomic surface area values ($\AA^2$) for heme bound to the ancestral glycosidase and the bound heme upon mutating to alanine in silico residues that block its access to the active site (Pro172, Asn173, Ile224, Leu226, Asn227 and Pro 272). As reference, the values for free heme are given in the last column.

| Atom | Ancestral | Ancestral with mutations to alanine | Free heme |
|---|---|---|---|
| NB | 0 | 4.9 | 4.9 |
| ND | 0 | 1.9 | 4.8 |
| C1 | 0 | 4.2 | 6.6 |
| C1 | 0 | 3.7 | 6.3 |
| C1 | 0 | 3.8 | 6.5 |
| C1 | 0 | 3.2 | 6.7 |
| C2 | 0 | 2.8 | 3 |
| C2 | 0.1 | 2.6 | 5.5 |
| C2 | 0 | 2.6 | 6 |
| C2 | 0 | 1.7 | 4.7 |
| C3 | 0 | 2.7 | 4.8 |
| C3 | 0 | 0 | 4.5 |
| C3 | 0 | 2.6 | 5.5 |
| C3 | 0 | 0.2 | 3.2 |
| C4 | 0 | 3.3 | 6.5 |
| C4 | 0 | 2.9 | 6.4 |
| C4 | 0 | 5.9 | 6.6 |
| C4 | 0 | 2.5 | 6 |
| CA | 0 | 20.6 | 20.6 |
| CA | 0 | 2.6 | 24.5 |
| CA | 5.6 | 12.8 | 25.2 |
| CA | 0.1 | 0.1 | 16.8 |
| CB | 0.1 | 4.8 | 20.4 |
| CB | 0.2 | 0.2 | 58.6 |
| CB | 10.7 | 20.9 | 58.5 |
| CB | 0 | 17 | 20.6 |
| CG | 0 | 2.1 | 7.8 |
| CG | 0 | 5.5 | 8.2 |
| CH | 0 | 3.9 | 8.4 |
| CH | 0 | 11.3 | 15.1 |
| CH | 0 | 5.7 | 12 |
| CH | 0.4 | 6.2 | 13.6 |
| CM | 0 | 37.3 | 54.6 |
| CM | 0 | 2.6 | 54 |
| CM | 0 | 4 | 52.4 |
| CM | 2.5 | 3.1 | 52.8 |
| NA | 0 | 4.2 | 5.1 |
| NC | 0 | 4.4 | 5.7 |
| O1 | 10.8 | 39.9 | 52.1 |
| O1 | 4.8 | 21 | 43.8 |
| O2 | 4.6 | 15.7 | 37.3 |
| O2 | 3.1 | 16.9 | 50 |
| FE | 0 | 5.2 | 5.2 |

**Table S9.** Data collection and refinement statistics (values in parentheses are for highest-resolution shell).

| Protein | ancestral | ancestral-heme |
|---|---|---|
| PDB ID | 6Z1H | 6Z1M |
| Space group | P 21 | P 21 |
| Unit cell | | |
| a, b, c (Å) | 52.26 80.67 97.81 | 58.93 89.49 141.12 |
| β (°) | 100.06 | 94.211 |
| ASU | 2 | 3 |
| Resolution (Å) * | 43.38 - 2.5 (2.59 - 2.5) | 52.87 - 2.45 (2.54 - 2.45) |
| $R_{merge}$ (%)* | 8.90 (105.30) | 80.96 (80.85) |
| $I/\sigma_I$ * | 10.9 (1.7) | 10.15 (1.44) |
| Completeness (%)* | 99.92 (99.93) | 99.02 (99.57) |
| Unique reflections* | 27829 (2775) | 53447 (5335) |
| Multiplicity | 7.4 (7.5) | 3.2 (3.2) |
| Wilson B-factor | 59.93 | 50.34 |
| CC(1/2) * | 0.999 (0.707) | 0.997 (0.718) |
| **Refinement** | | |
| $R_{work}/R_{free}$ (%) | 19.59 / 24.10 | 17.55 / 22.09 |
| No. atoms | 6601 | 11026 |
| Protein | 6546 | 10676 |
| Ligands | 21 | 194 |
| Solvent | 34 | 156 |
| B-factor ($Å^2$) | 76.63 | 62.81 |
| R.m.s deviations | | |
| Bond lengths (Å) | 0.003 | 0.005 |
| Bond angles ($^0$) | 0.65 | 0.73 |
| Ramachandran (%) | | |
| Favored | 96.09 | 96.14 |
| Outliers | 0.13 | 0 |

*Statistics for the highest-resolution shell are shown in parentheses.

## Supplementary FIgures

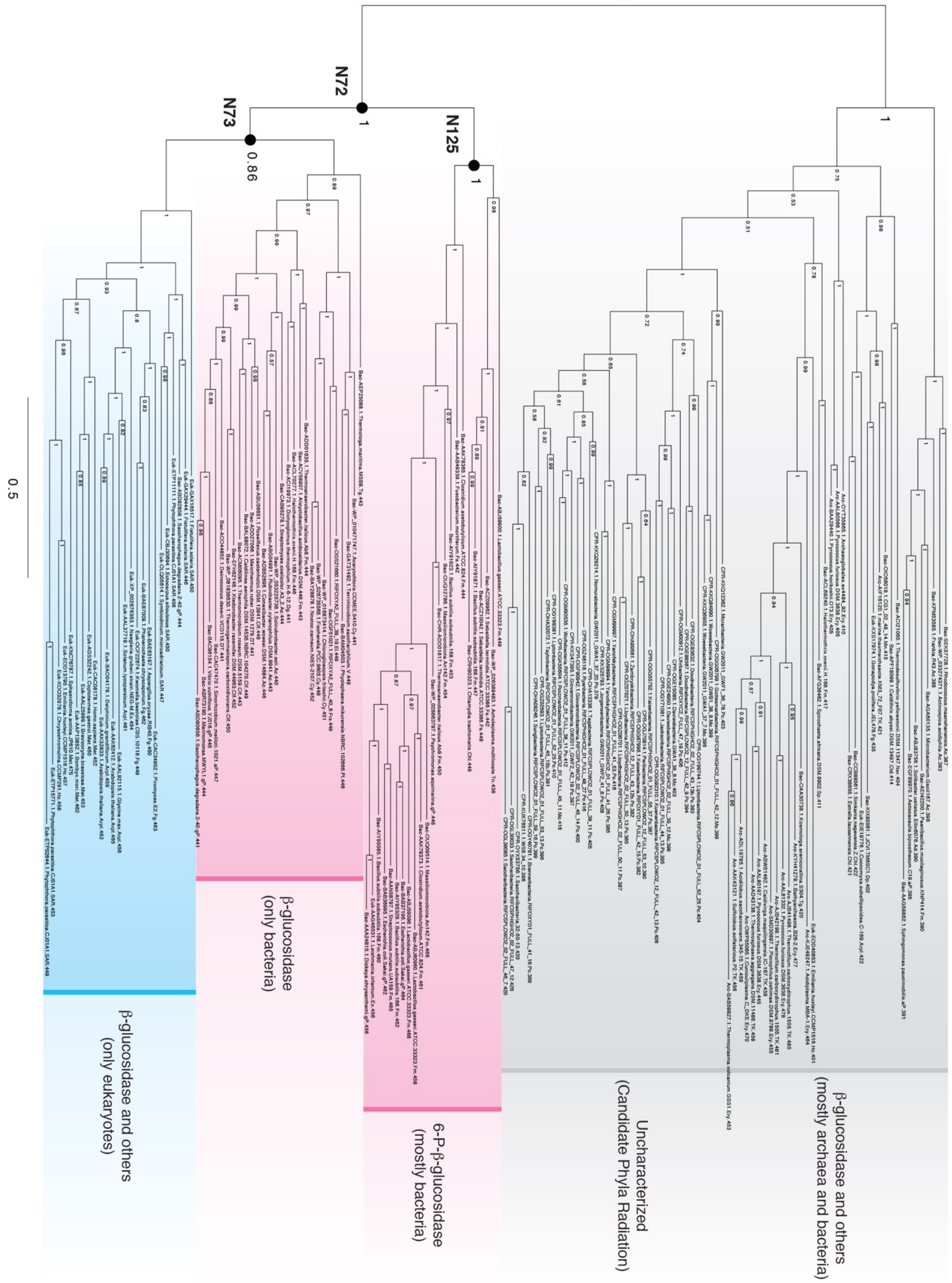


**Figure S1.** Bayesian analysis of family 1 glycosidases (GH1) protein sequences with sequence annotations. The annotation includes the accession number, the taxonomical information (domain name, phylum name and species name) and the sequence length. Three black dots indicate three ASR nodes (N72, N73 and N125). Scale bar represents 0.5 amino acid replacements per site per unit evolutionary time. Abbreviations: Ac =

Actinobacteria; aP = $\alpha$-Proteobacteria; Arc = Archaea; Arpl = Archaeplastida; Asg = Asgard group; Bac = Bacteria; Bc = Bacteroidetes; bP = $\beta$-Proteobacteria; Chl = Chlamydiae; Cld = Calditrichaeota (Caldithrix); Clf = Cloroflexi; CPR = Candidate Phyla Radiation; Cy = Cyanobacteria; Euk = Eukaryotes; Ex = Excavates; Fg = Fungi; Fm = Firmicutes; Frs = Fraserbacteria; Dg = Dictyoglomi; dP = $\delta$-Proteobacteria; Dp = Dependentiae (TM6); DPN = DPANN group; DT = Deinococcus-Thermus; Ery = Euryarchaeota; Fs = Fusobacteria; gP = $\gamma$-Proteobacteria; Hc = Hacrobia; Mc = Microgenomates; Met = Metazoa; Mn = Marinimicrobia; Nsr = Nitrospirae; Pc = Parcubacteria; Pl = Planctomycetes; V = Verrucomicrobia; SAR = SAR group; Sp = Spirochaetes; Tg = Thermotogae; TK = TACK group; Tn = Tenericutes.

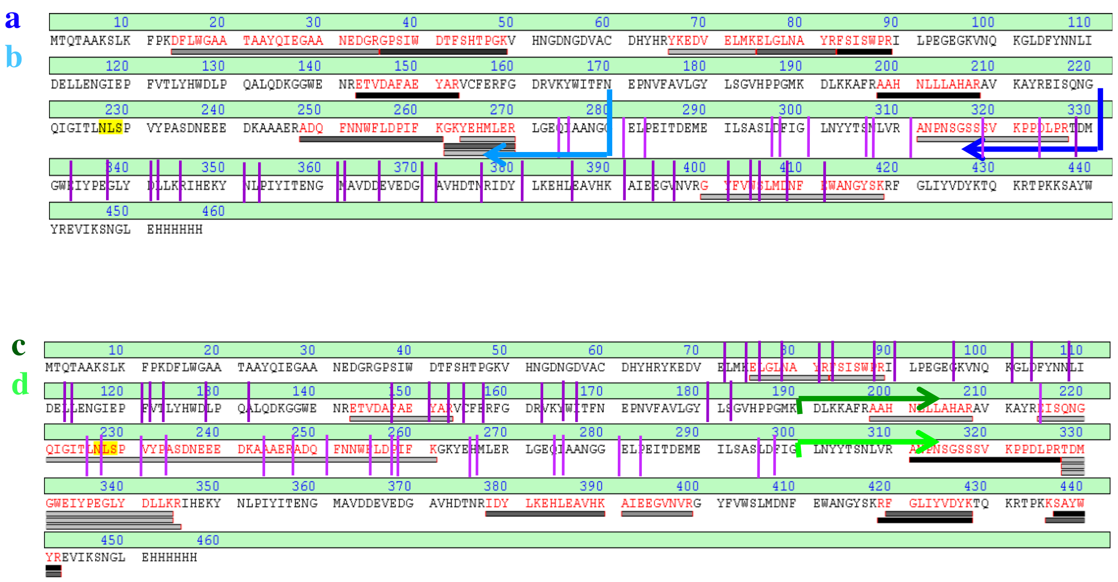


**Figure S2.** Estimation of the location of thermolysin cleavage sites from mass spectrometry and peptide-mapping fingerprinting. Potential thermolysin restriction sites are shown by vertical purple lines. Four thermolysin fragments were studied (a, b, c, and d: see left for color code). Fragment masses were determined by MALDI and their sequences were investigated using peptide mapping finger-printing and MALDI-TOF/TOF. Sequences for several sub-fragments (shown) could be thus determined and the length of the original fragments could be assessed. Fragments a and b extend approximately from the amino terminus to the dark and light blue arrows in the upper panel. Fragments c and d extend approximately from the dark and light green arrows in the lower panel to the carboxyl terminus. Comparison with the restriction sites allows a determination of the plausible thermolysin cleavage sites, as shown in Figure 2C of the main text.

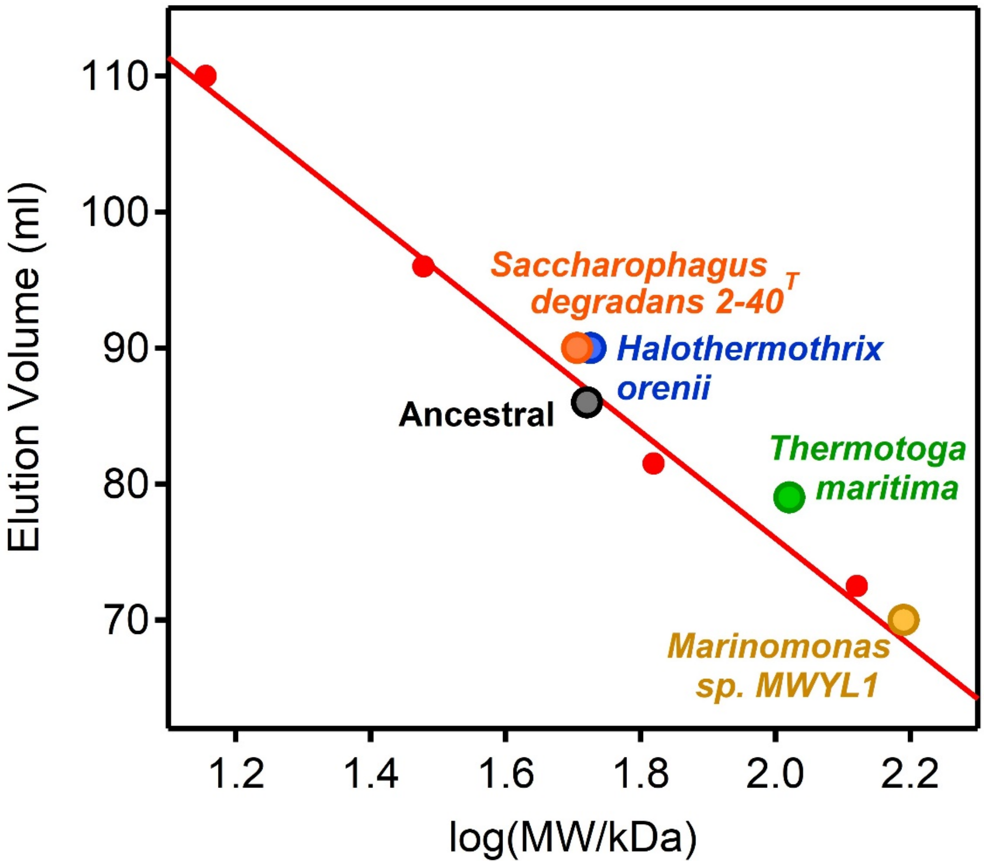


**Figure S3.** Assessment of association state of modern and ancestral glycosidases through gel filtration chromatography (HiLoad 16/600 Superdex 200 pg GE Healthcare). The molecular mass (MW) was estimated by the calibration curve of elution volume vs. log (MW). The protein markers used were: bovine serum albumin (monomer: 66 kDa, dimer: 132 kDa), deoxyribonuclease I from bovine pancreas (30.1 kDa) and lysozyme (14.3 kDa). The ancestral glycosidase and the modern glycosidases from *Saccharophagus degradans and Halothermothrix orenii* are monomers. The modern glycosidases from *Thermotoga maritima* is a dimer and the modern glycosidase from *Marinomonas sp. MWTL1* is a trimer.

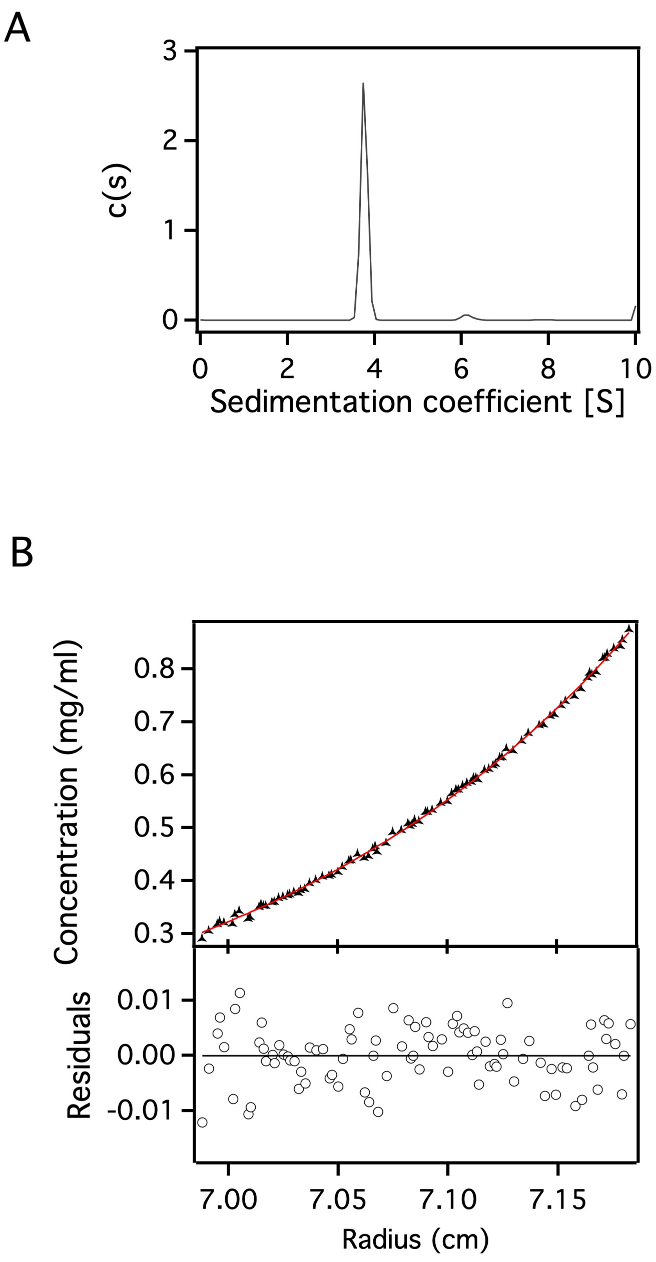


**Figure S4.** Assessing the association state of the ancestral glycosidase through analytical ultracentrifugation. A) Sedimentation velocity assay showing the sedimentation coefficient distribution c(s) corresponding to 0.24 mg/ml of purified protein. Peak at 3.7S is compatible with a globular monomer with the theoretical mass derived from the sequence. B) Sedimentation equilibrium assay. Upper panel: concentration gradient of experimental data (triangles) are presented together with best-fit analysis assuming protein monomer (red line). Lower panel: Difference between experimental data and estimated values for a protein monomer model (residuals in mg/mL). A molecular mass of 52900±192 Da is obtained, which is sufficiently close to the theoretical monomer mass calculated from the sequence (52542.79 Da) to rule out the dimeric and higher association states.

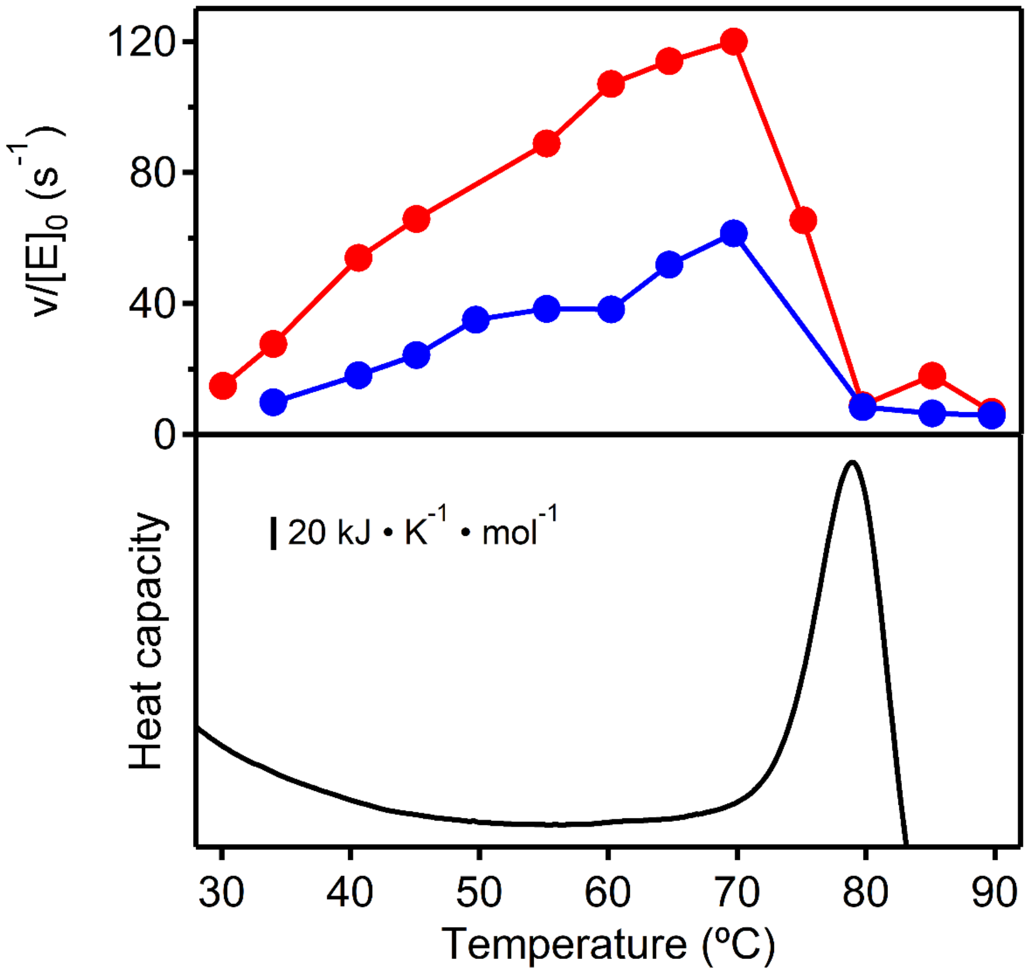


**Figure S5.** Determination of the optimum temperature for the modern glycosidase from *Halothermothrix orenii* using two different substrates 4-nitrophenyl-β-D-glucopyranoside (red) and 4-nitrophenyl-β-D-galactopyranoside (blue). The lower panel shows a differential scanning calorimetry profile for the enzyme under the same buffer conditions. Clearly, the activity drop observed at high temperature (upper panel) corresponds to the denaturation of the protein, as seen in the lower panel.

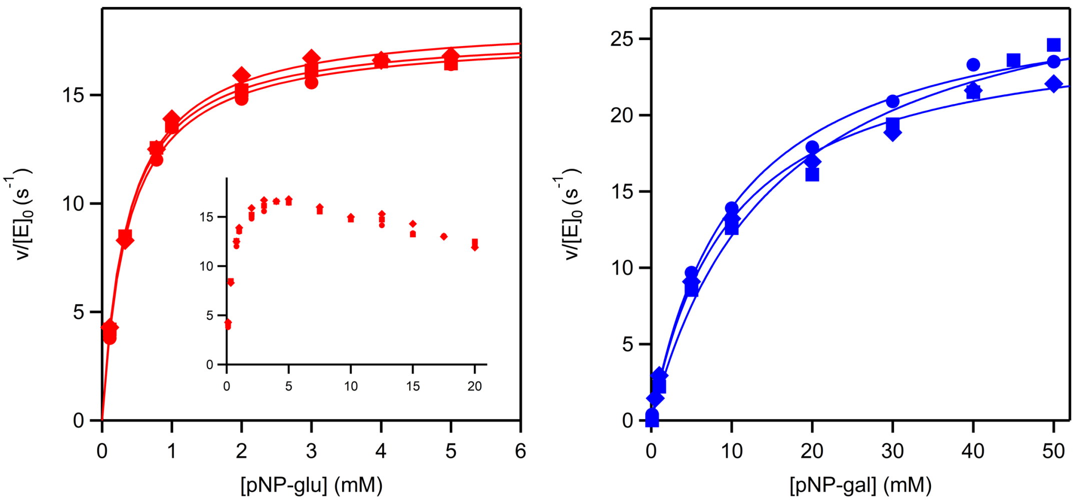


**Figure S6**. Michaelis plots of rate versus substrate concentration at pH 7 and 25 ºC for the hydrolysis of 4-nitrophenyl-β-D-glucopyranoside and 4-nitrophenyl-β-D-galactopyranoside catalyzed by the modern glycosidase from *Thermotoga maritima.* The different data points correspond to the 3 experimental replicates performed for each substrate, involving two different protein preparations in each case. The lines are the best fits of the Michaelis-Menten equation (see Tables S3 and S4 for the values derived from the fits). It is well known that glycosidase catalysis often shows kinetic complexities at high substrate concentrations, due to phenomena such as transglycosylation, inhibition by substrate or allosteric activation[8]. As a result of these complexities, Michaelis-Menten saturation kinetics are sometimes not observed. Here Michaelis-Menten saturation kinetics is not observed for 4-nitrophenyl-β-D-glucopyranoside in a wide concentration range (see inset in panel at the left). Therefore, only the data up to 6 mM have been used for the determination of the catalytic parameters from the fitting of the Michaelis-Menten equation.

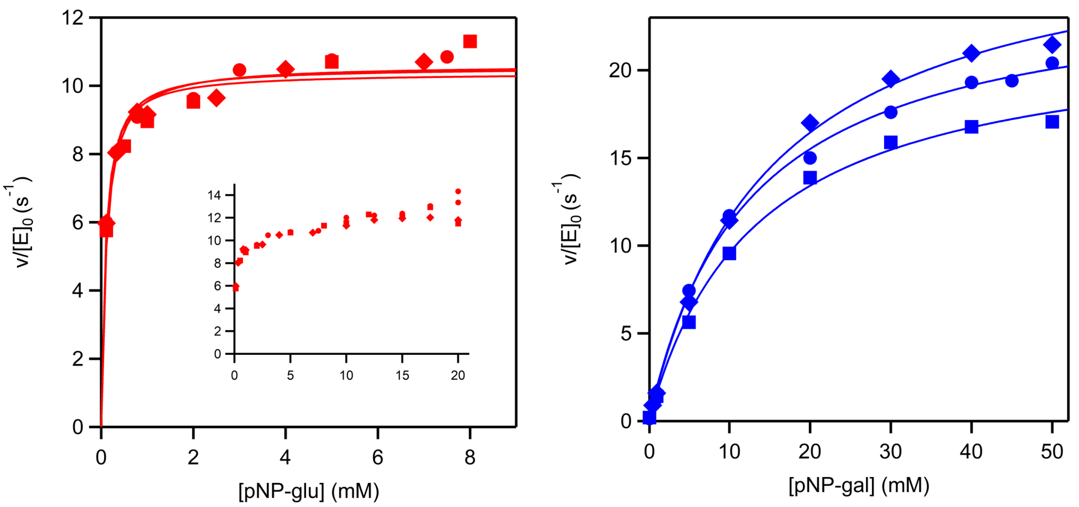


**Figure S7**. Michaelis plots of rate versus substrate concentration at pH 7 and 25 ºC for the hydrolysis of 4-nitrophenyl-β-D-glucopyranoside and 4-nitrophenyl-β-D-galactopyranoside catalyzed by the modern glycosidase from *Marinomonas sp.* (strain MWYL1). The different data points correspond to the 3 experimental replicates performed for each substrate, involving two different protein preparations in each case. The lines are the best fits of the Michaelis-Menten equation (see Tables S2 and S3 for the values derived from the fits). It is well known that glycosidase catalysis often shows kinetic complexities at high substrate concentrations, due to phenomena such as transglycosylation, inhibition by substrate or allosteric activation[8]. Here Michaelis-Menten saturation kinetics is not for 4-nitrophenyl-β-D-glucopyranoside in a wide concentration range (see inset in panel at the left). Therefore, only the data up to 8 mM have been used for the determination of the catalytic parameters from the fitting of the Michaelis-Menten equation.

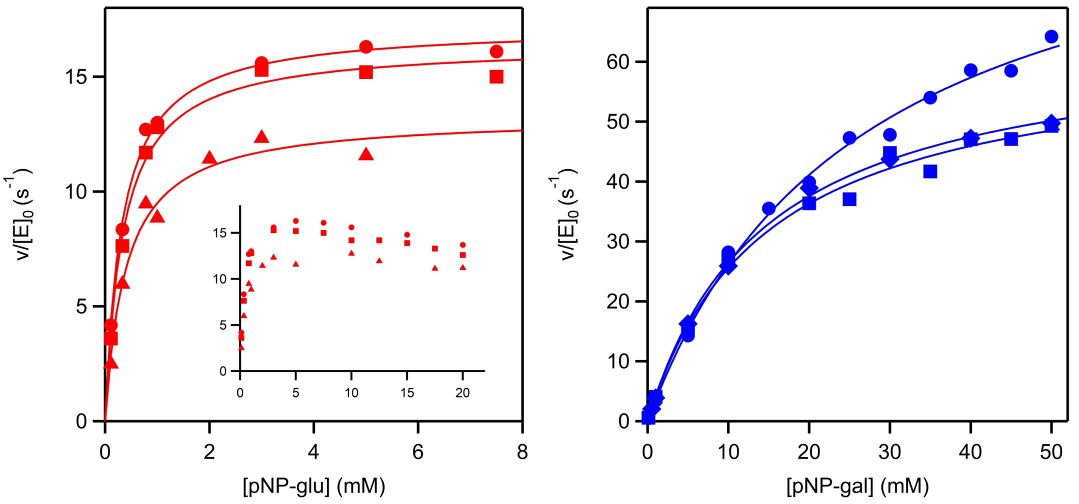


**Figure S8**. Michaelis plots of rate versus substrate concentration at pH 7 and 25 ºC for the hydrolysis of 4-nitrophenyl-β-D-glucopyranoside and 4-nitrophenyl-β-D-galactopyranoside catalyzed by the modern glycosidase from *Halothermothrix orenii.* The different data points correspond to the 3 experimental replicates performed for each substrate, involving two different protein preparations in each case. The lines are the best fits of the Michaelis-Menten equation (see Tables S2 and S3 for the values derived from the fits). It is well known that glycosidase catalysis often shows kinetic complexities at high substrate concentrations, due to phenomena such as transglycosylation, inhibition by substrate or allosteric activation[8]. Here Michaelis-Menten saturation kinetics is not for 4-nitrophenyl-β-D-glucopyranoside in a wide concentration range (see inset in panel at the left). Therefore, only the data up to 8 mM have been used for the determination of the catalytic parameters from the fitting of the Michaelis-Menten equation.

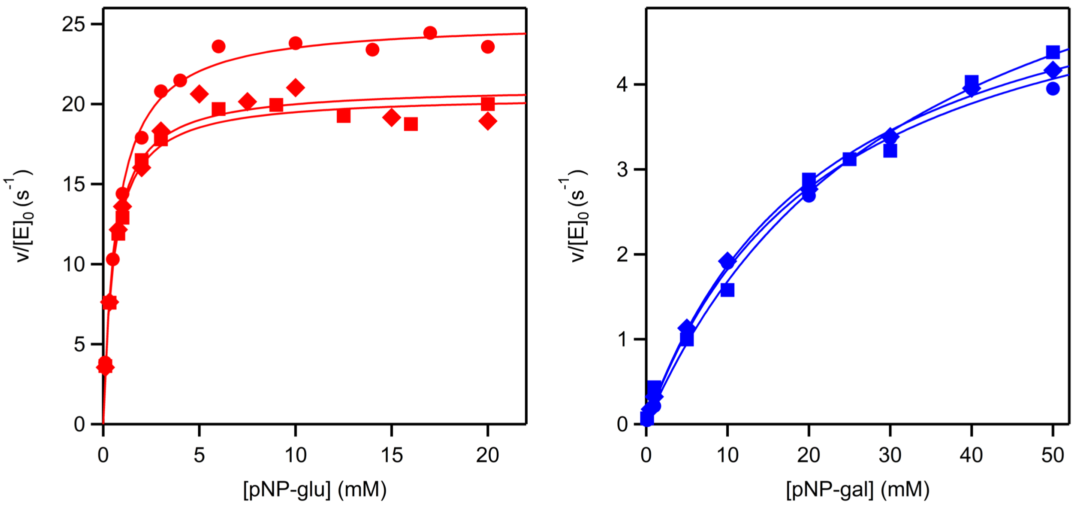


**Figure S9**. Michaelis plots of rate versus substrate concentration at pH 7 and 25 ºC for the hydrolysis of 4-nitrophenyl-β-D-glucopyranoside and 4-nitrophenyl-β-D-galactopyranoside catalyzed by the modern glycosidase from *Saccharophagus degradans.* The different data points correspond to the 3 experimental replicates performed for each substrate, involving two different protein preparations in each case. The lines are the best fits of the Michaelis-Menten equation (see Tables S2 and S3 for the values derived from the fits).

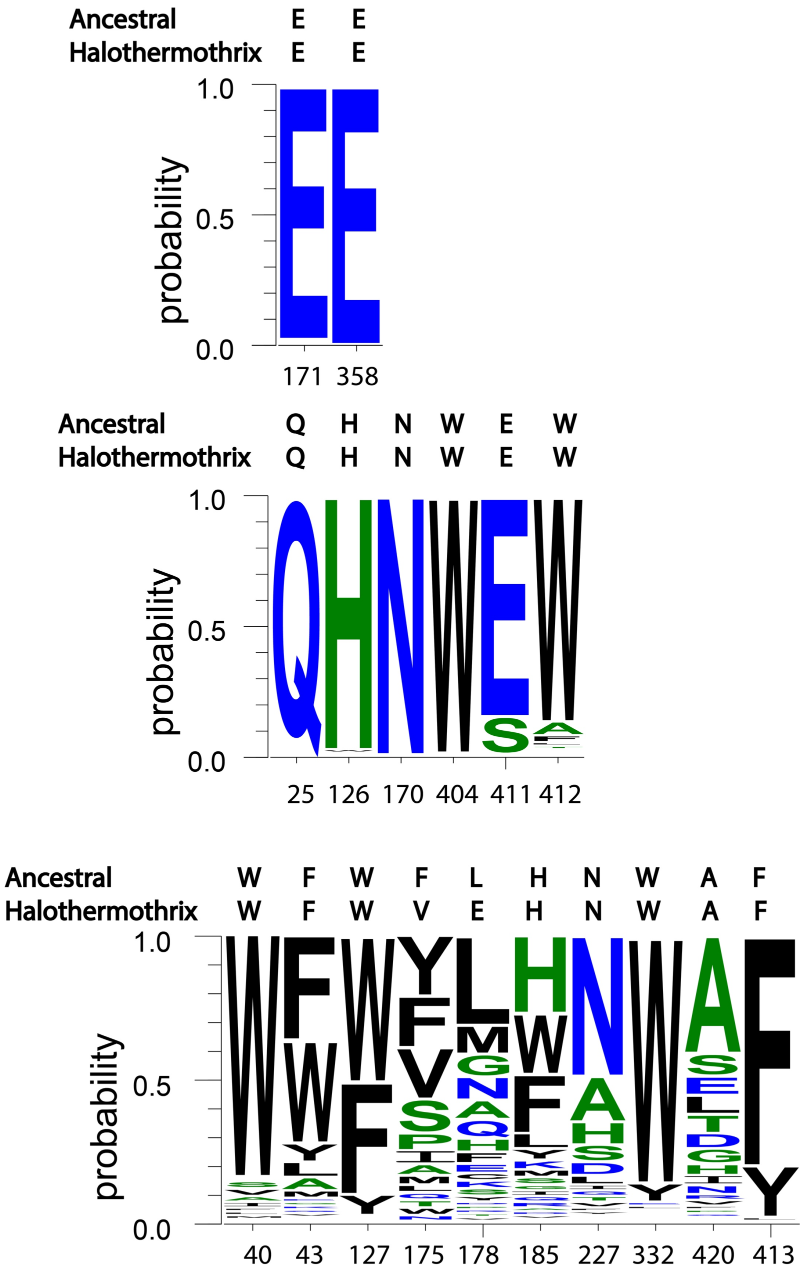


**Figure S10.** Statistics of residue occupancy at critical active site positions in the set of modern glycosidases used as starting point for ancestral sequence reconstruction (see also Table S4). The graphics shown refer to the catalytic carboxylic acids (upper), the positions involved in the binding of the glycone moiety of the substrate (middle) and the positions involved in the binding of the aglycone moiety of the substrate (lower). The sequences of the ancestral glycosidase and the modern glycosidase from *Halothermothrix orenii* are also given. Glycosidases are known to be somewhat specific for the glycone moiety of the substrate and much less specific for the aglycone moiety, which is reflected in lower residue conservation at the protein residues involved in aglycone binding.

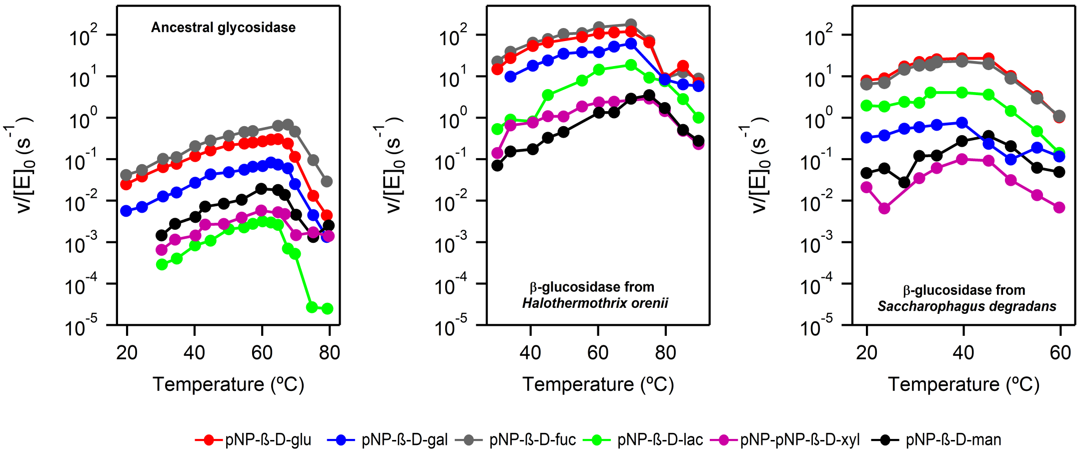


**Figure S11.** Profiles of activity versus temperature for the ancestral glycosidase (left) and the two modern glycosidases from *Halothermothrix orenii* and *Saccharophagus degradans* using the following substrates: *4*-nitrophenyl-β-D-glucopyranoside, *4*-nitrophenyl-β-D-galactopyranoside, *4*-nitrophenyl-β-D-fucopyranoside, *4*-nitrophenyl-β-D-lactopyranoside, *4*-nitrophenyl-β-D-xylopyranoside and *4*-nitrophenyl-β-D-mannopyranoside. Activity values were derived from determination of *p*-nitrophenolate after 10 minutes incubation of 1 mM substrate with the enzyme, as described in Methods.

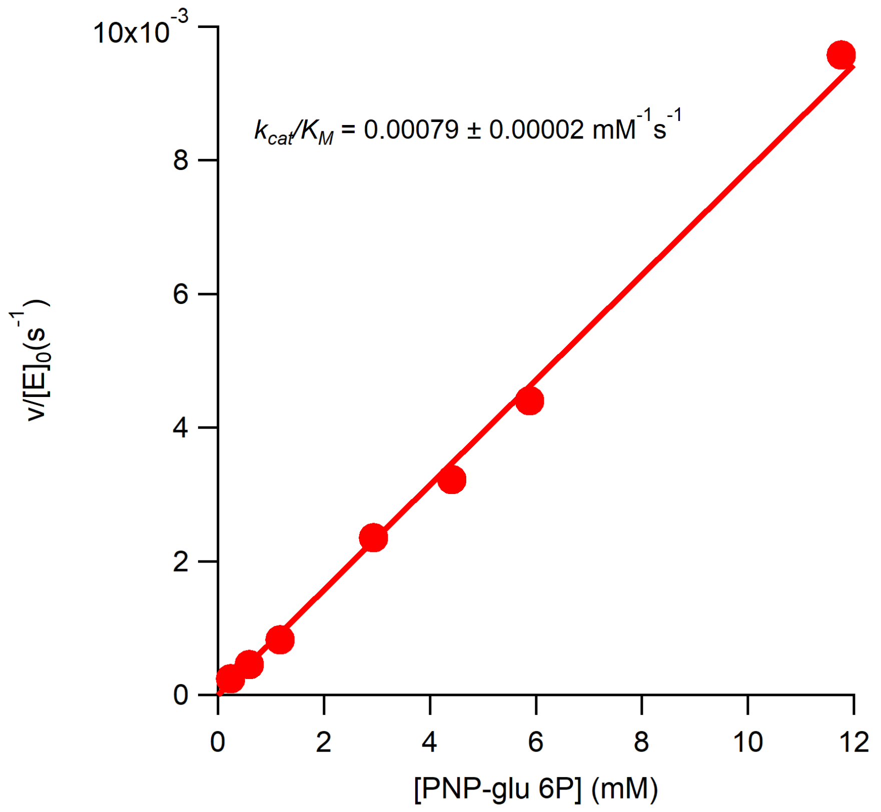


**Figure S12**. Michaelis plot of rate versus substrate concentration for the hydrolysis of *4*-nitrophenyl-β-D-glucopyranoside-6-phosphate catalysed by the ancestral glycosidase. No curvature is observed in the plot and, therefore, only the value for the catalytic efficiency can be derived from the experimental data. This value is about ~40 times lower than the catalytic efficiency with the corresponding non-phosphorylated substrate.

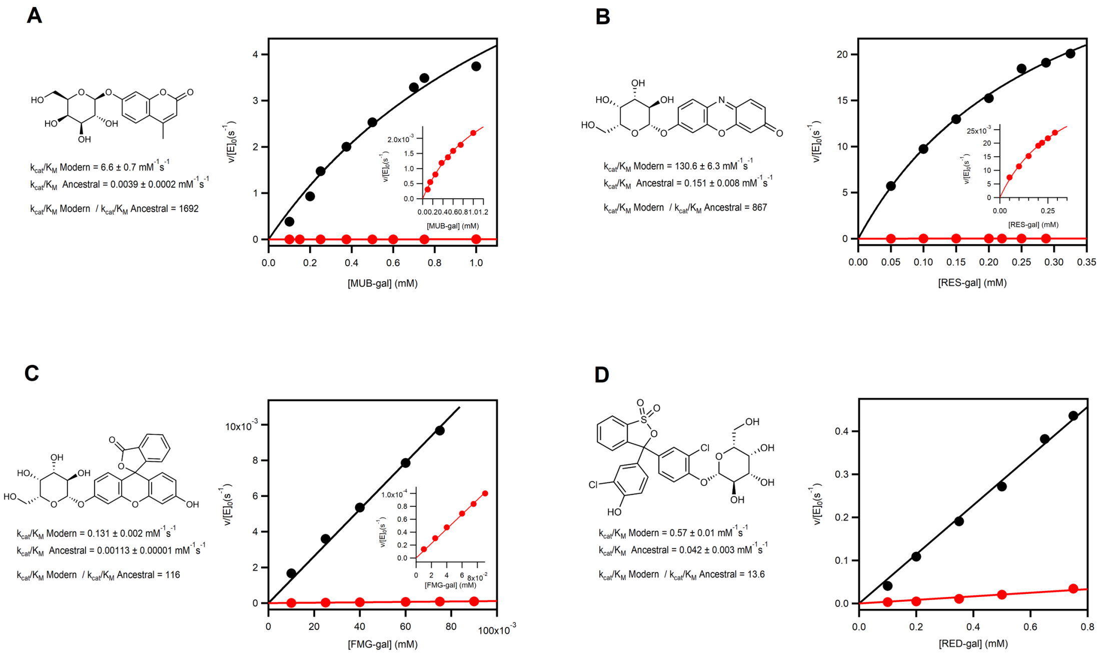


**Figure S13**. Michaelis plots of rate *versus* substrate concentration for the hydrolysis of the indicated substrates catalyzed by the ancestral glycosidase (red and insets) and the modern glycosidase from *Halothermothrix orenii* (black). All the substrates used are β-D-galactopyranosides with a large aglycone moiety. Catalytic efficiencies derived from the fits of the Michaelis-Menten equation are shown.

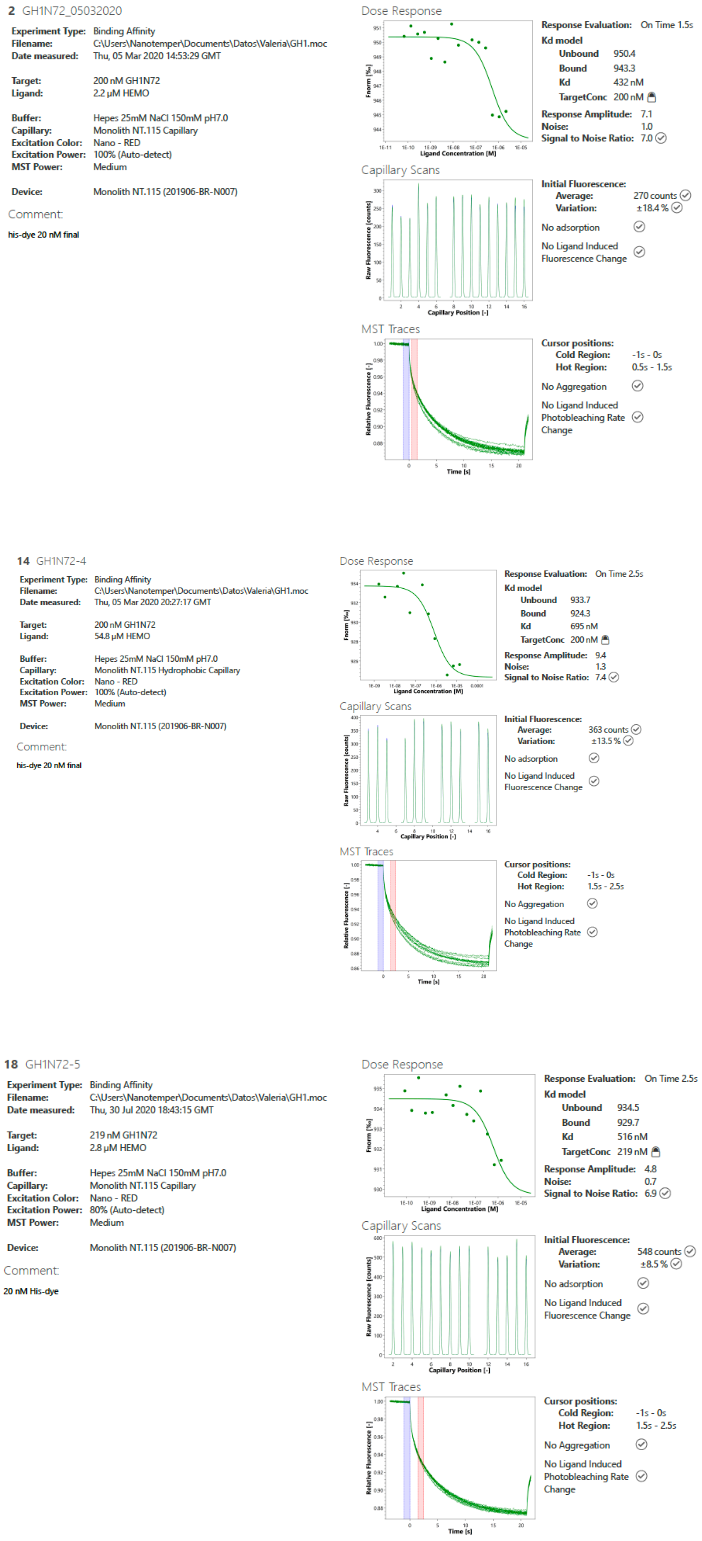


**Figure S14.** Relevant experimental data plots and validation reports for the quantification of heme binding to the ancestral glycosidase using microscale thermophoresis. Note that information for three replicate experiments is provided. Since bound heme is monomeric, while heme in solution at neutral pH has a tendency to associate, it is possible that the reported dissociation constants are overestimates (*i.e.,* binding could be even tighter than suggested by these values).

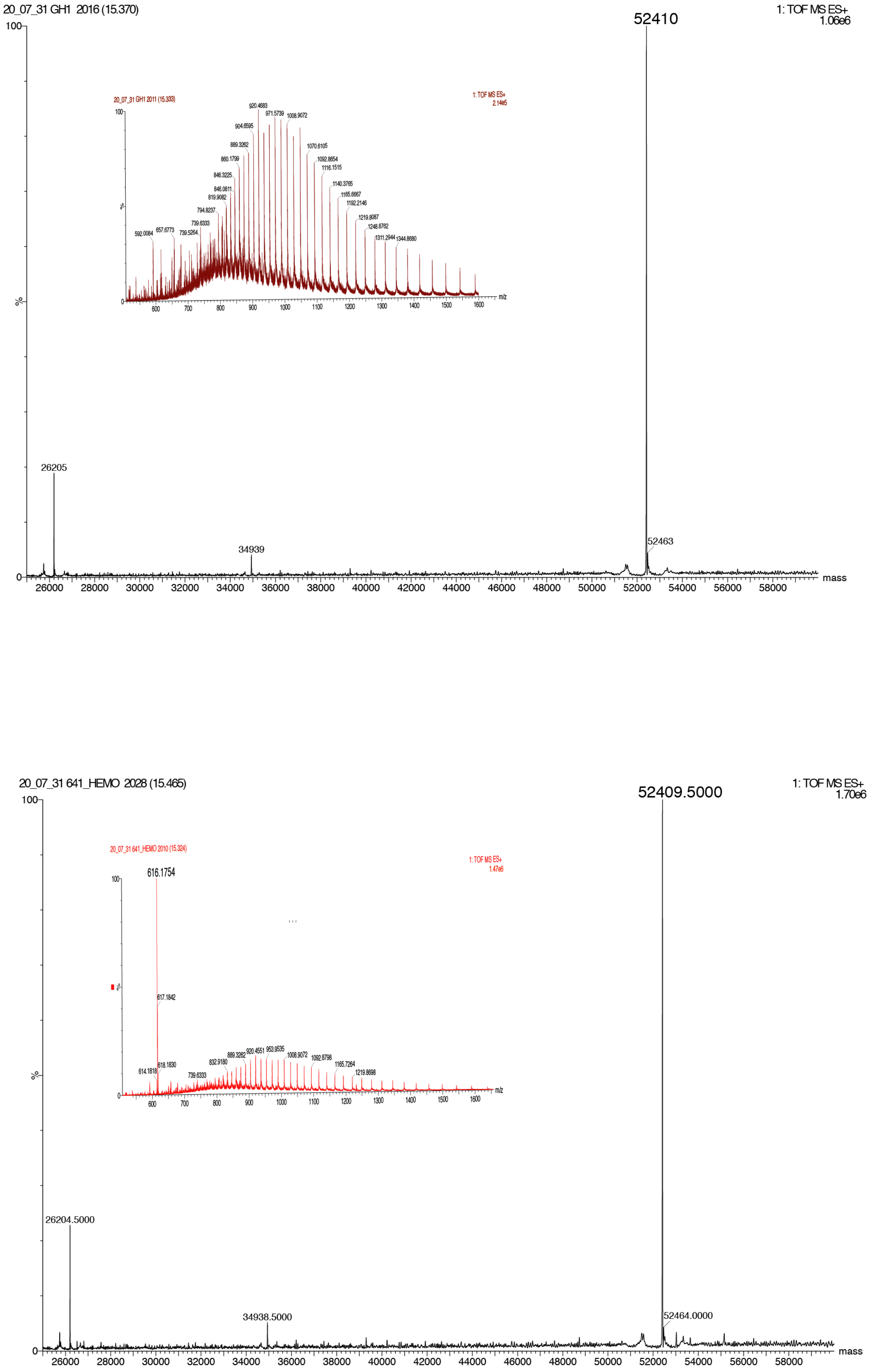


**Figure S15.** Mass spectra after UPLC elution of samples of the ancestral glycosidase originally without (upper panel) and with (lower panel) bound heme. The protein peak is apparent at a mass near the theoretical value calculated from the amino acid sequence (52542.79 Da). The insets correspond to the low mass range where heme is

expected to appear. A peak of mass essentially close to that expected for heme (651.94 Da) is observed only in the lower panel. The intensity of the heme peak is qualitatively similar to that of the protein peak, as expected from the 1:1 binding stoichiometry.

A

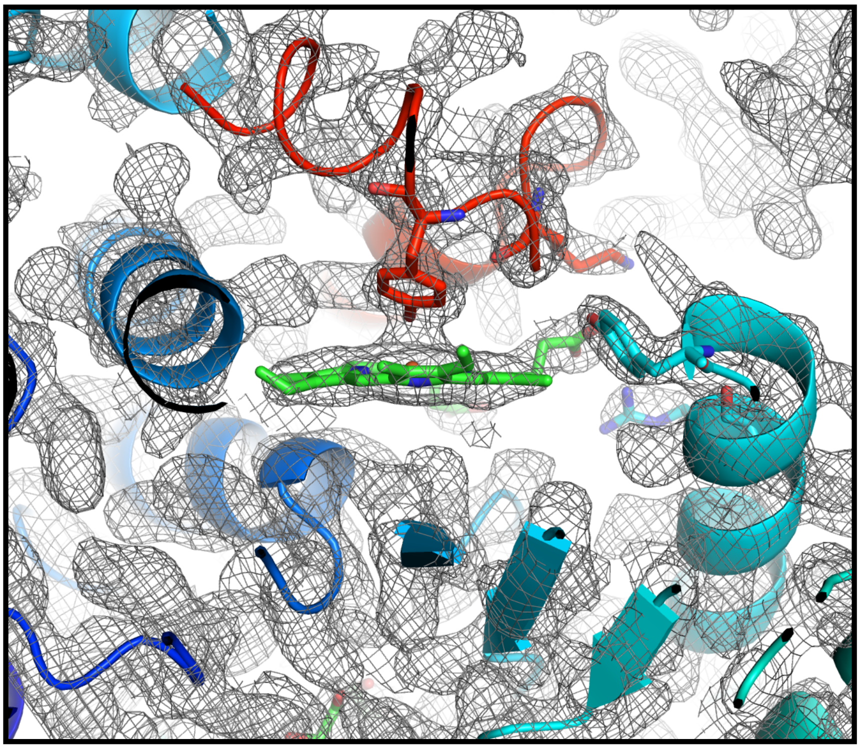

B

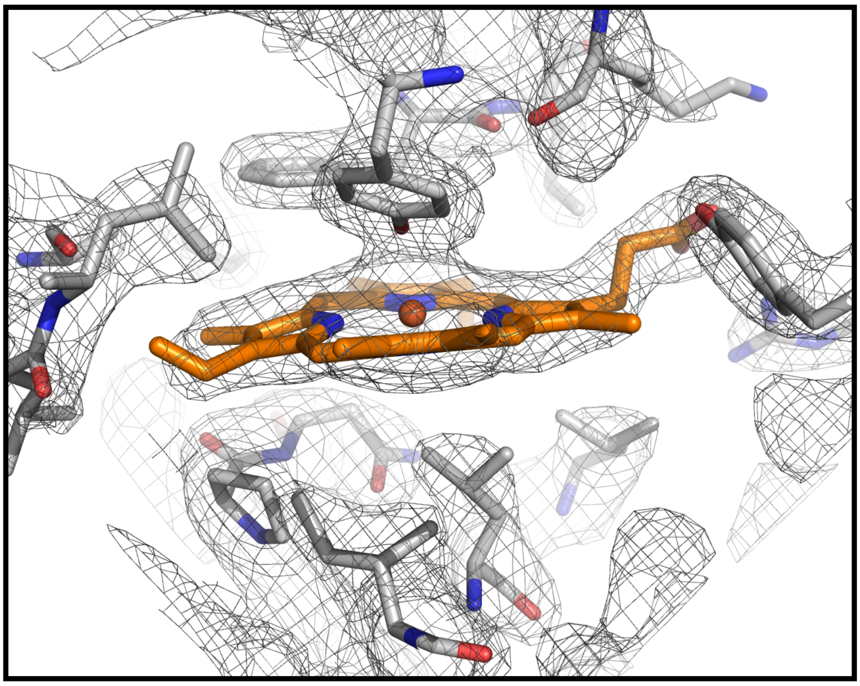

**Figure S16.** (A) Structure of the ancestral glycosidase showing the bound heme group into the well-defined |2Fo-Fc| electron density map contoured at 1 σ. The four amino acids involved in hydrogen bonds (See Figure 7A) are shown as sticks. B) Blow-up showing all residue participating in binding the heme group.

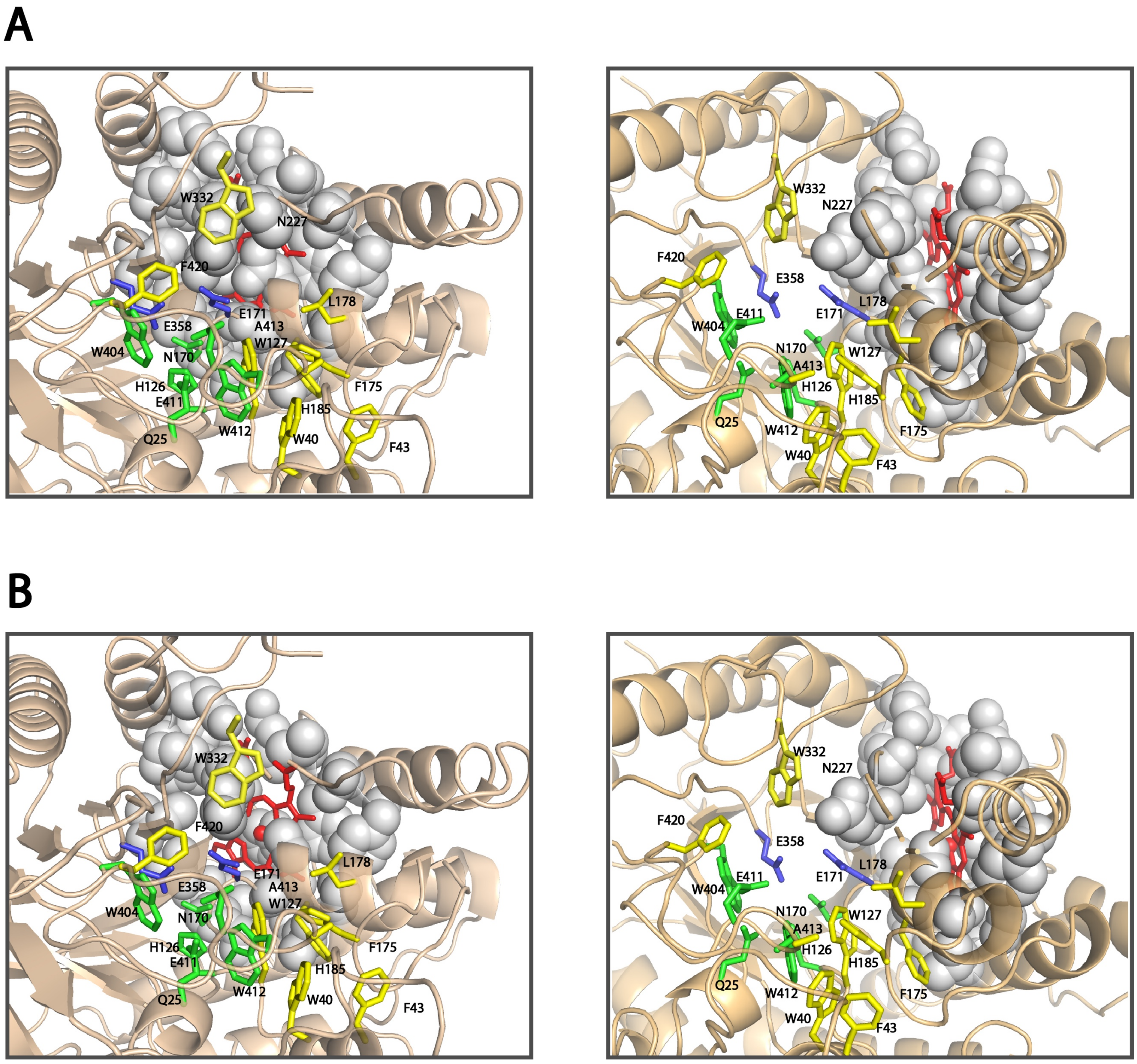


**Figure S17**. (A) View of the active site of the ancestral protein showing the catalytic carboxylic acid residues (blue) and the residues involved in binding of the glycone (yellow) and aglycone (green) parts of the substrate molecule. The residues that block the connection of the heme group with the active site are shown with van der Waals spheres and colored in grey. (B) Same as in C, but the residues blocking the connection of the heme with the active site have been computationally mutated to alanine, in such a way that now the iron of the heme group can be seen at the bottom of the active site in the chosen view.

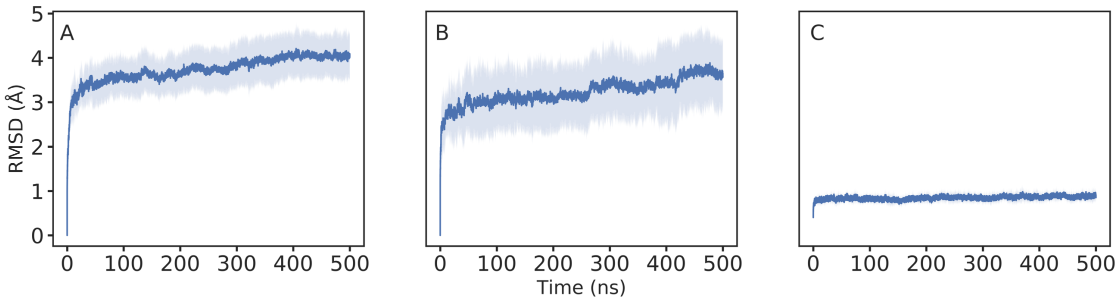


**Figure S18.** The root mean square deviations (RMSD, Å) of all backbone atoms of the (A) ancestral glycosidase without heme bound, (B) ancestral glycosidase with heme bound and (C) modern glycosidase from *Halothermothrix orenii* over ten individual 500 ns MD simulations per system (i.e. 5 μs cumulative simulation time per system). The center for the error band (solid blue line) shows the average RMSD obtained per system at a given time frame for each replica, while the standard deviations are given as the shaded areas on each plot. n = 10 independent simulations.

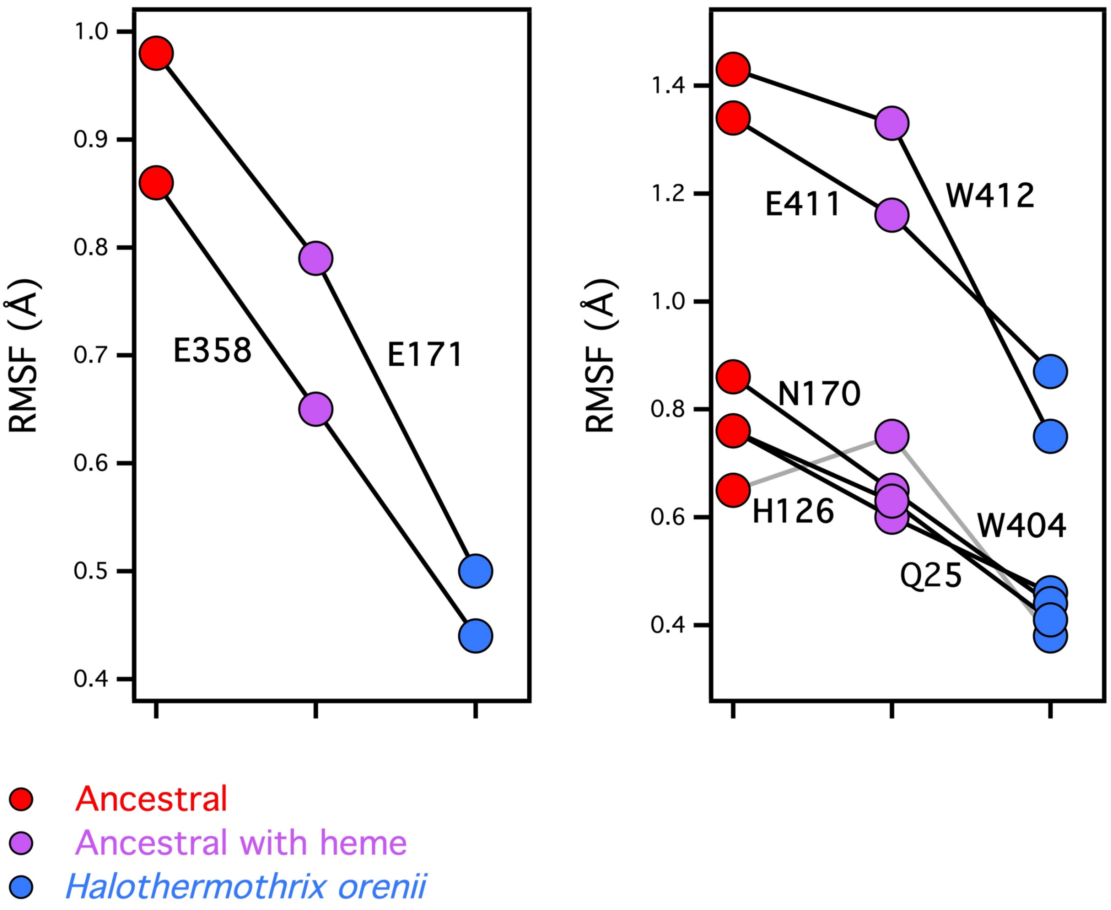


**Figure S19.** Root mean square deviations of all backbone atoms of the catalytic carboxylic acid residues (left) and residues involved in the binding of the glycone moiety of the substrate (right). Values are shown for the ancestral glycosidase with and without heme as well as for the modern glycosides from *Halothermothrix orenii*. This figure is a complement to Figure 3 in the main text, further details about the analysis are provided in the caption to Figure 3. See also Figures 2D and S10 for more information about the residues selected for this figure.

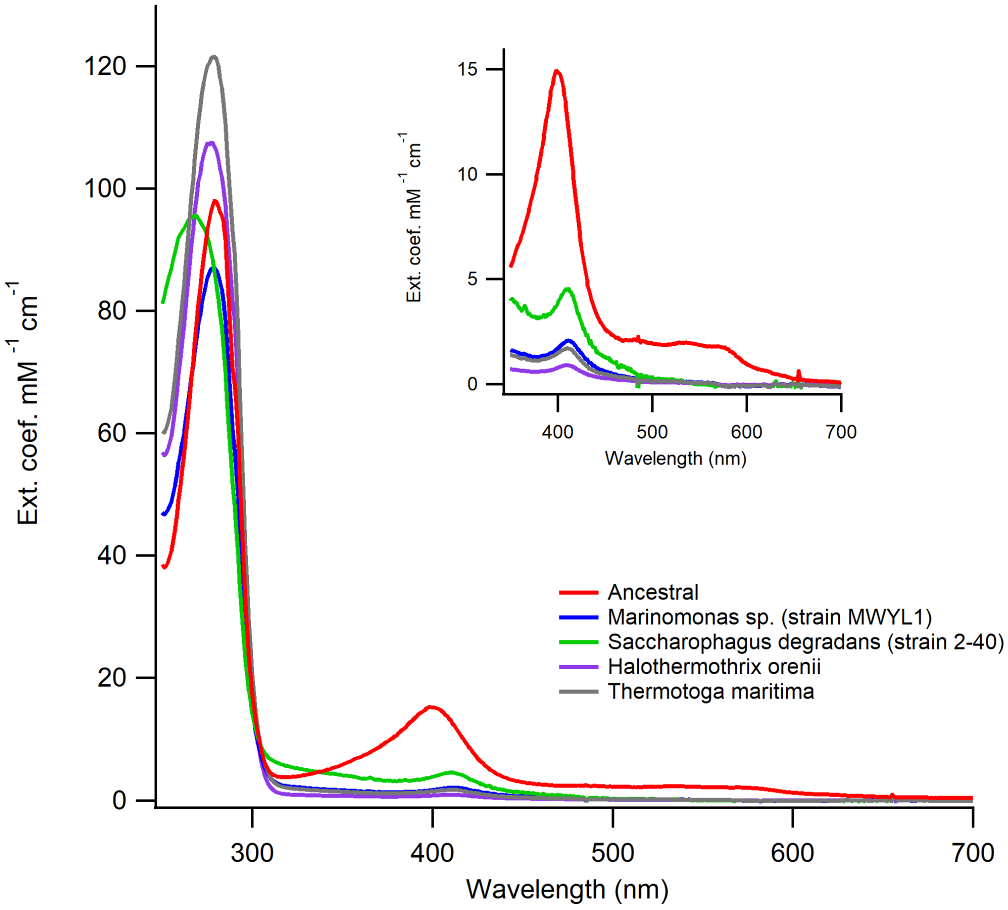


**Figure S20.** UV-VIS spectra of protein preparations of modern glycosidases showing the protein absorption band at 280 nm and the Soret heme band at about 400 nm. The inset is a blow-up of the Soret band region. For comparison, data for the ancestral glycosidase (corresponding to the reconstruction at node 72) are also included. In all preparations, 0.4 mM 5-aminolevulinic acid (the metabolic precursor of heme) was added to the culture medium and the protein was purified by Ni-NTA affinity chromatography and further passage through a PD10 column.

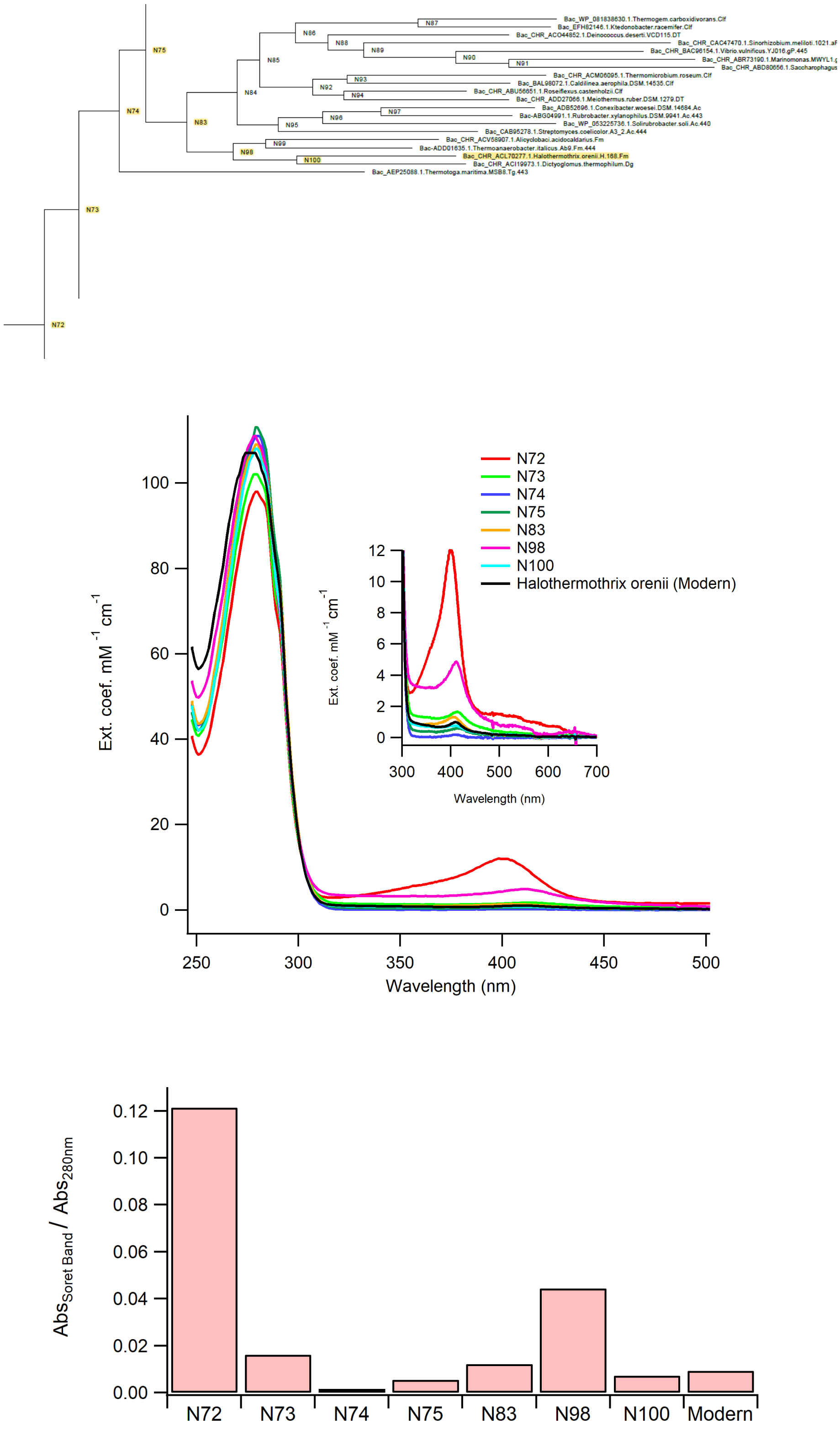


**Figure S21**. UV-VIS spectra of protein preparations corresponding to reconstructions of nodes in the line of descent leading from the ancestral glycosidase at node 72 to the modern glycosidase from *Halothermothrix orenii*. For comparison, data for the modern glycosidase are included. In all preparations, 0.4 mM 5-aminolevulinic acid (the metabolic precursor of heme) was added to the culture medium and the protein was

purified by Ni-NTA affinity chromatography and further passage through a PD10 column. Upper panel: section of the phylogenetic tree used as a basis for sequence reconstruction (Figure S1) highlighting the nodes studied here. Middle panel: UV-VIS spectra showing the protein absorption band at 280 nm and the Soret heme band at about 400 nm. The inset is a blow-up of the Soret band region. Lower panel: ratio of absorbance at the maximum of the heme Soret band to the absorbance at the maximum of the protein aromatic absorption band.

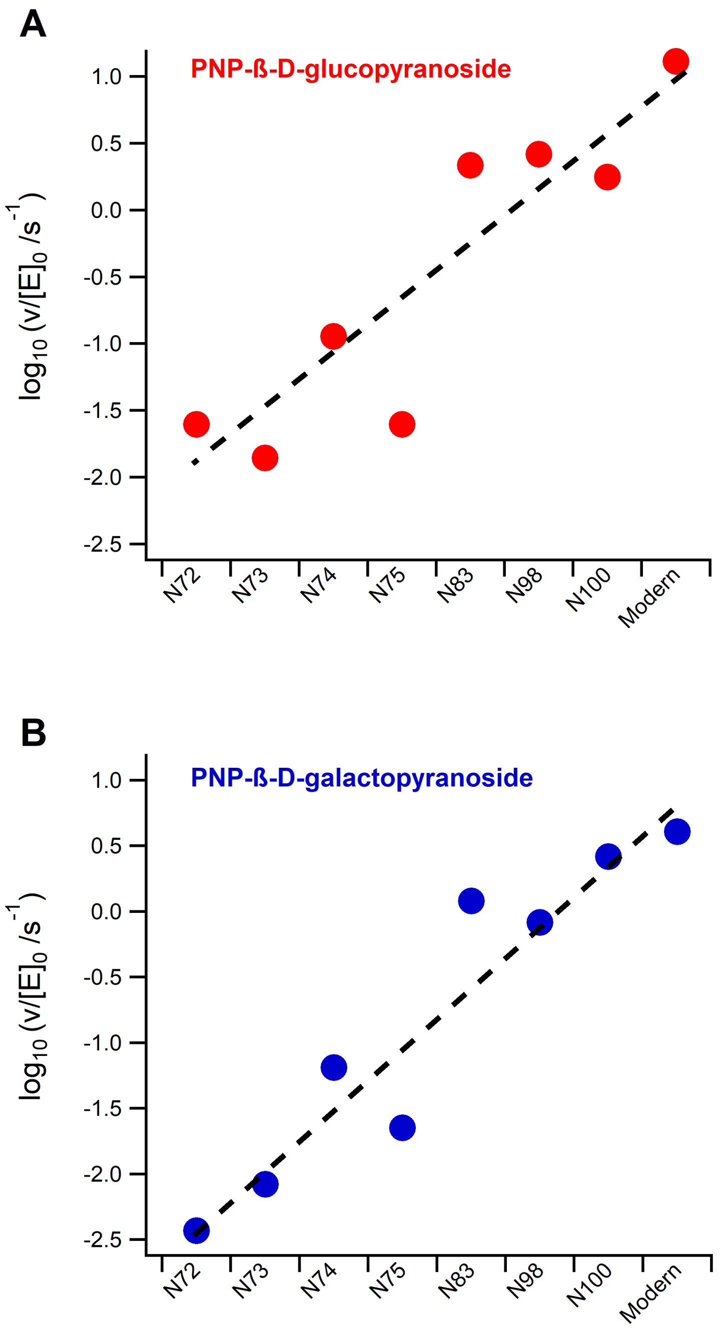


**Figure S22.** Glycosidase activity of protein preparations corresponding to reconstructions of nodes in the line of descent leading from the ancestral glycosidase at node 72 to the modern glycosidase from *Halothermothrix orenii* (labelled as modern). Assays were performed at pH 7 and 25 ºC with 1 mM concentration of *4*-nitrophenyl-β-D-glucopyranoside or *4*-nitrophenyl-β-D-galactopyranoside. The dashed lines are only meant to guide the eye.

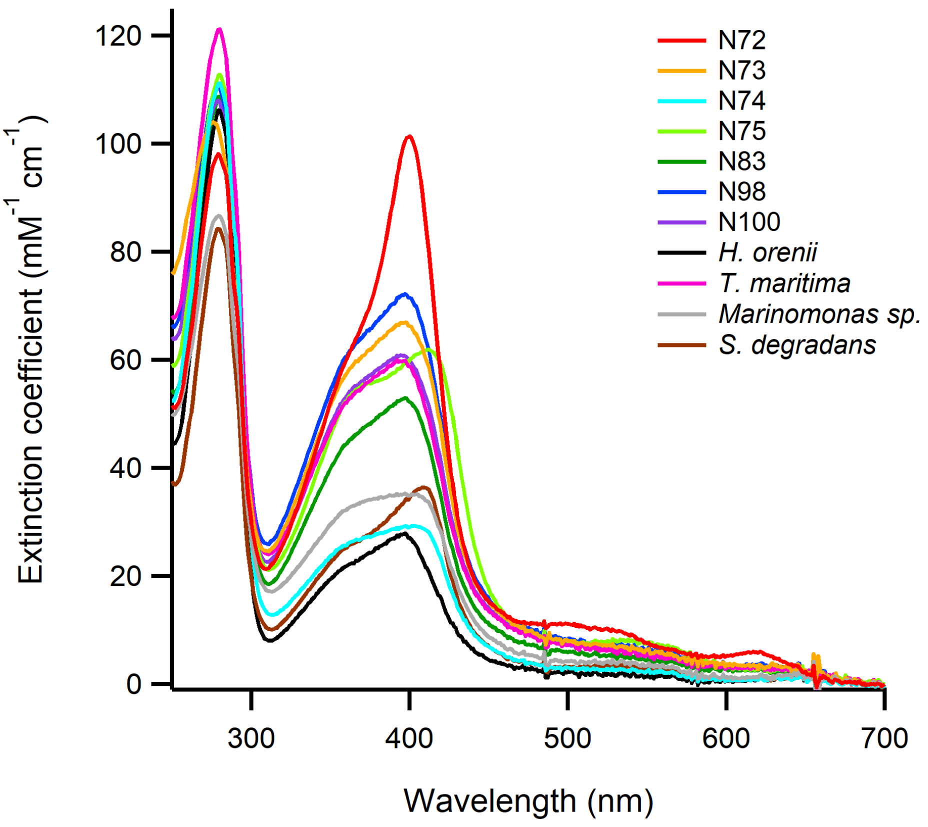


**Figure S23.** UV-VIS spectra of protein preparations corresponding to reconstructions of nodes in the line of descent leading from the ancestral glycosidase at node 72 to the modern glycosidase from *Halothermothrix orenii* (see Figure 8 in the main text and Figure S21). For comparison, data for three additional modern glycosidases are also included. In all preparations, heme-free protein samples at ~5 μM concentration were incubated with for 1 hour at pH 7 with a 5-fold excess of heme and free heme was removed by exclusion chromatography (2 passages through PD10 columns) before recording the UV-VIS spectra.

**N73**

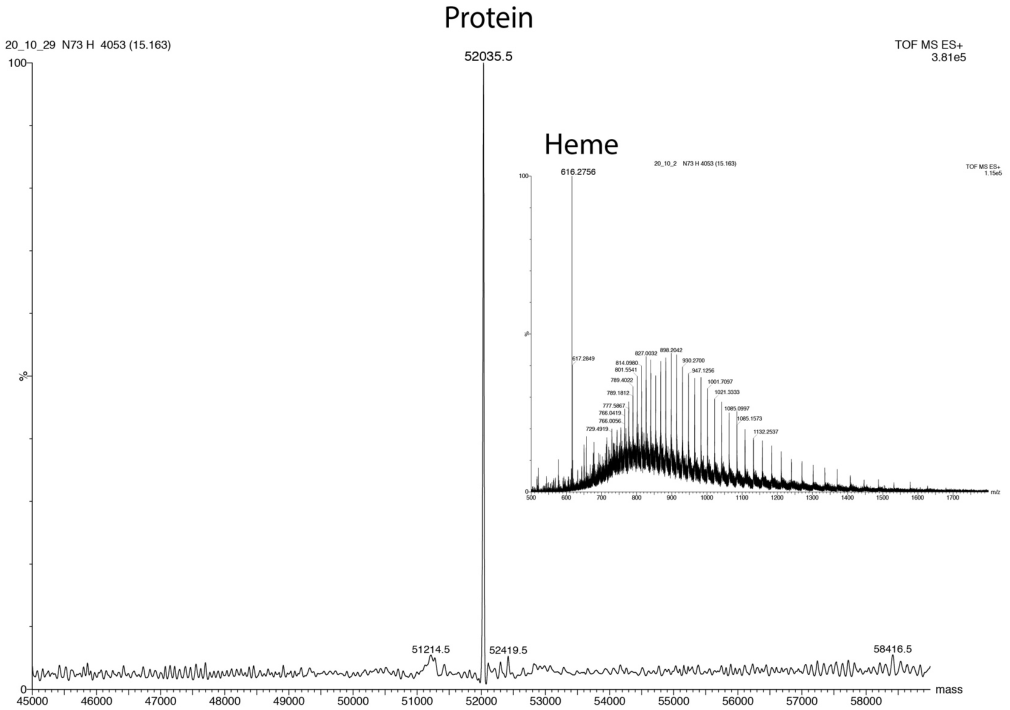


**Figure S24.** Mass spectra after UPLC elution of samples of the ancestral glycosidase corresponding to node 73 (Figure 8A and upper panel in Figure S21) with bound heme (see legends to Figures 8 and S21 for details). The protein peak is apparent at a mass near the theoretical value calculated from the amino acid sequence. The insets correspond to the low mass range where heme is expected to appear. A peak of mass close to that expected for heme is observed. This peak was not present in preparations of the protein without bound heme.

**N74**

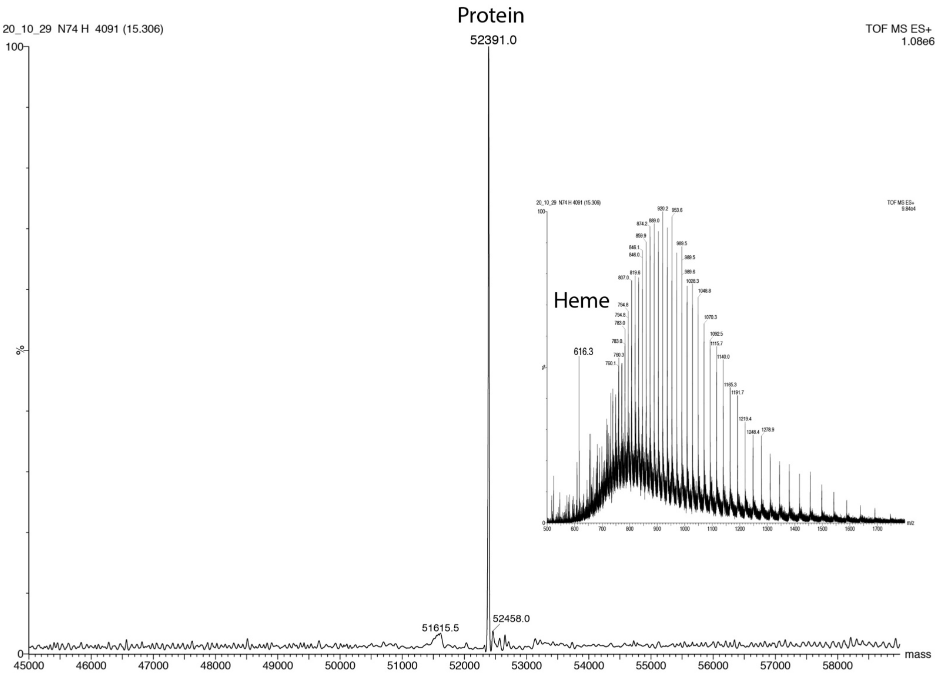


**Figure S25.** Mass spectra after UPLC elution of samples of the ancestral glycosidase corresponding to node 74 (Figure 8A and upper panel in Figure S21) with bound heme (see legends to Figures 8 and S21 for details). The protein peak is apparent at a mass near the theoretical value calculated from the amino acid sequence. The insets correspond to the low mass range where heme is expected to appear. A peak of mass close to that expected for heme is observed. This peak was not present in preparations of the protein without bound heme.

**N75**

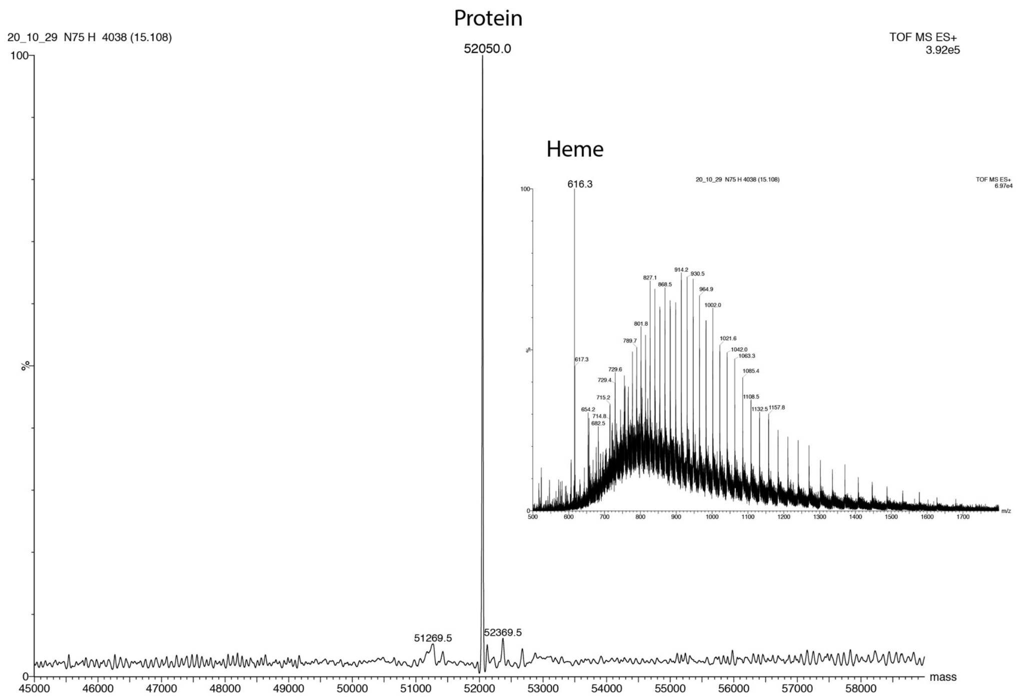


**Figure S26.** Mass spectra after UPLC elution of samples of the ancestral glycosidase corresponding to node 75 (Figure 8A and upper panel in Figure S21) with bound heme (see legends to Figures 8 and S21 for details). The protein peak is apparent at a mass near the theoretical value calculated from the amino acid sequence. The insets correspond to the low mass range where heme is expected to appear. A peak of mass close to that expected for heme is observed. This peak was not present in preparations of the protein without bound heme.

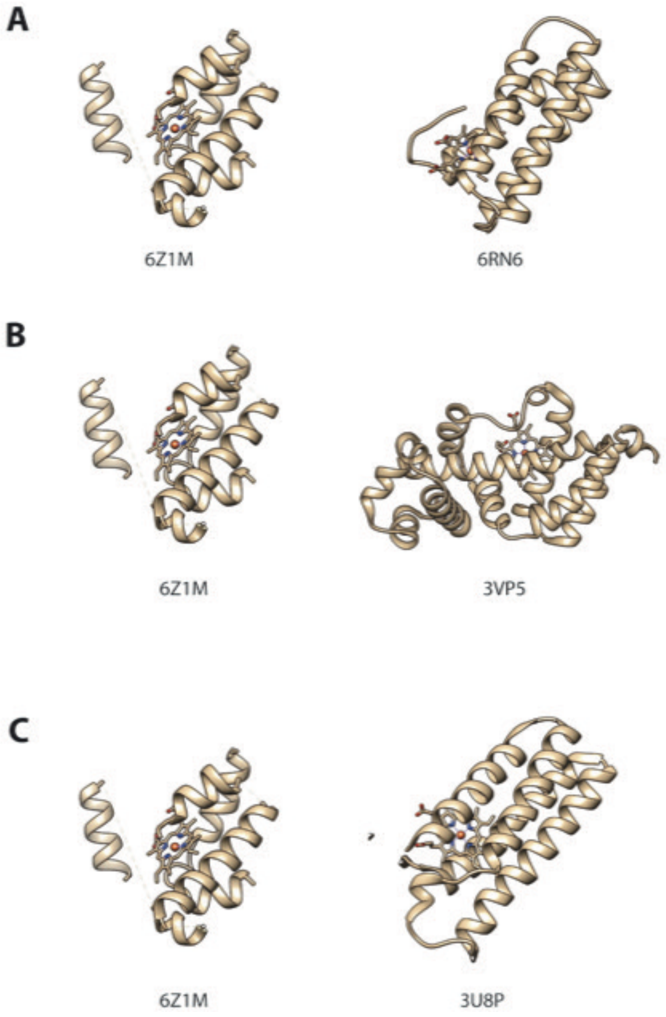


**Figure S27**. Comparison of the $\alpha$-helix structure around the bound heme in the ancestral glycosidase (left) with the results of a DALI search of the Protein Data Bank. The $\alpha$-helices involved in heme binding in our ancestral glycosidase were used as query for a structural alignment search. This resulted in 222 hits with RMSD values ranging from 1.6 to 11.4 Å. Only 3 of those hits had bound heme (shown here as A; B and C). The corresponding structures in the heme binding region are shown at the right. The corresponding structural alignments had RMSD ~4 Å and Z scores of 2 or higher.

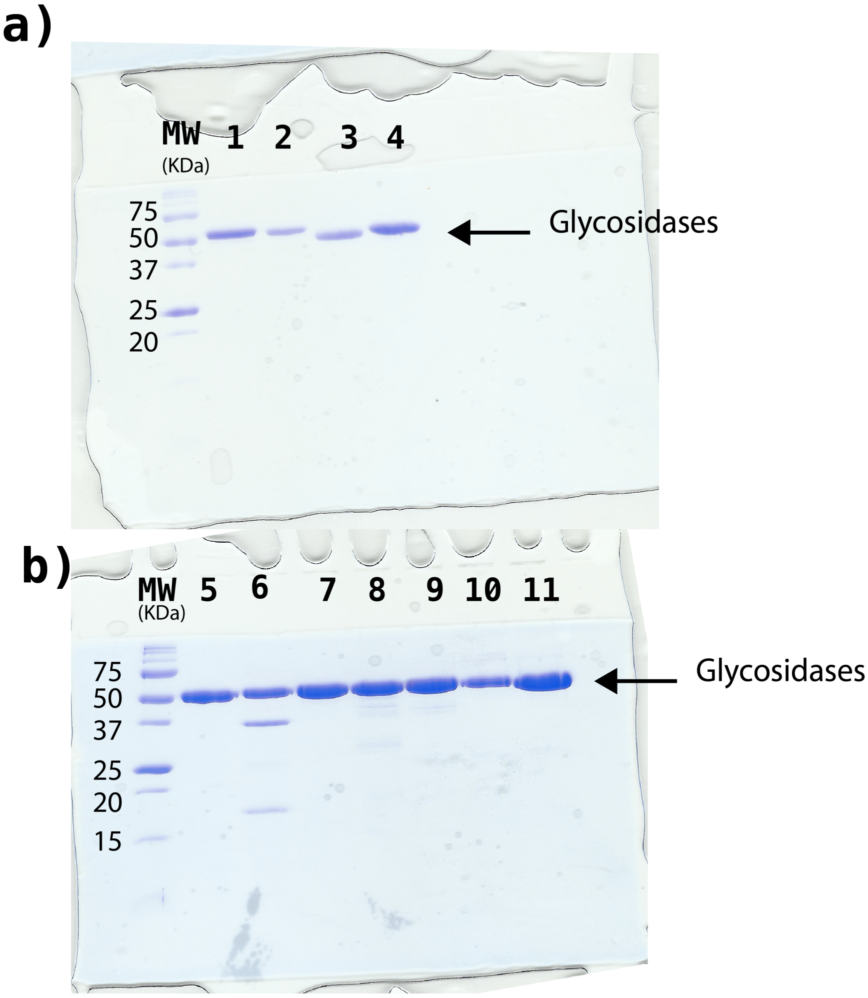


**Figure S28.** SDS gel electrophoresis of preparations of modern and ancestral proteins studied in this work. Code is as follows: MK: molecular weight markers. 1: *Halothermothrix orenii.* 2: *Thermotoga maritima.* 3: *Saccharophagus degradans (strain 2-40T).* 4: *Marinomonas sp. (strain MWYL1).* 5: N72. 6: N73. 7: N74. 8: N75. 9: N83. 10: N98. 11: N100 (Figure 8 in the main text and upper panel in Figure S21). Densitometry quantification indicated the the purity of the protein samples was in the range 93%-98%. Three independent experiments were performed with similar results.

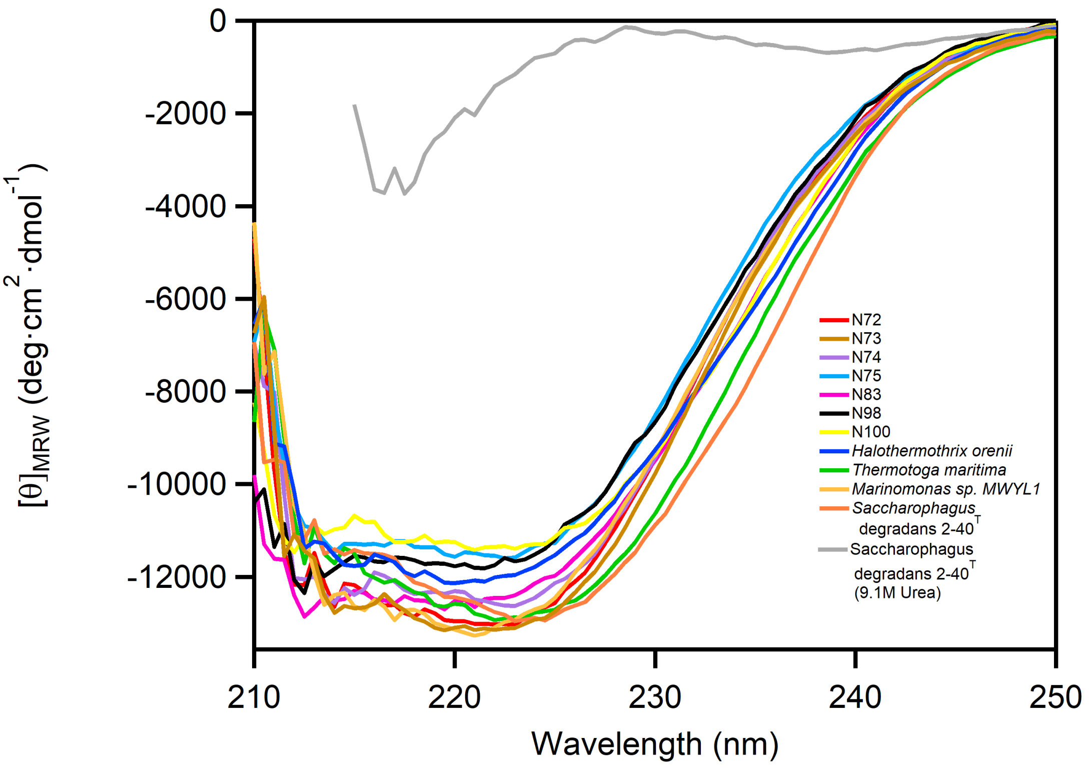


**Figure S29.** Circular dichroism spectra of modern and ancestral proteins studied in this work (see Figure 8 in the main text and upper panel of Figure S21 for the identification of the ancestral nodes). Conditions were 50 mM HEPES, pH 7.0, protein concentration within the 0.2-0.6 mg/mL range and a 1 mm pathlength cuvette. An average of 30 scans was performed in each case. Blank subtraction was always carried out prior to mean residue ellipticity calculation. For comparison, the spectra of *Saccharophagus degradans* glycosidase unfolded in 9.1 M urea is also reported.

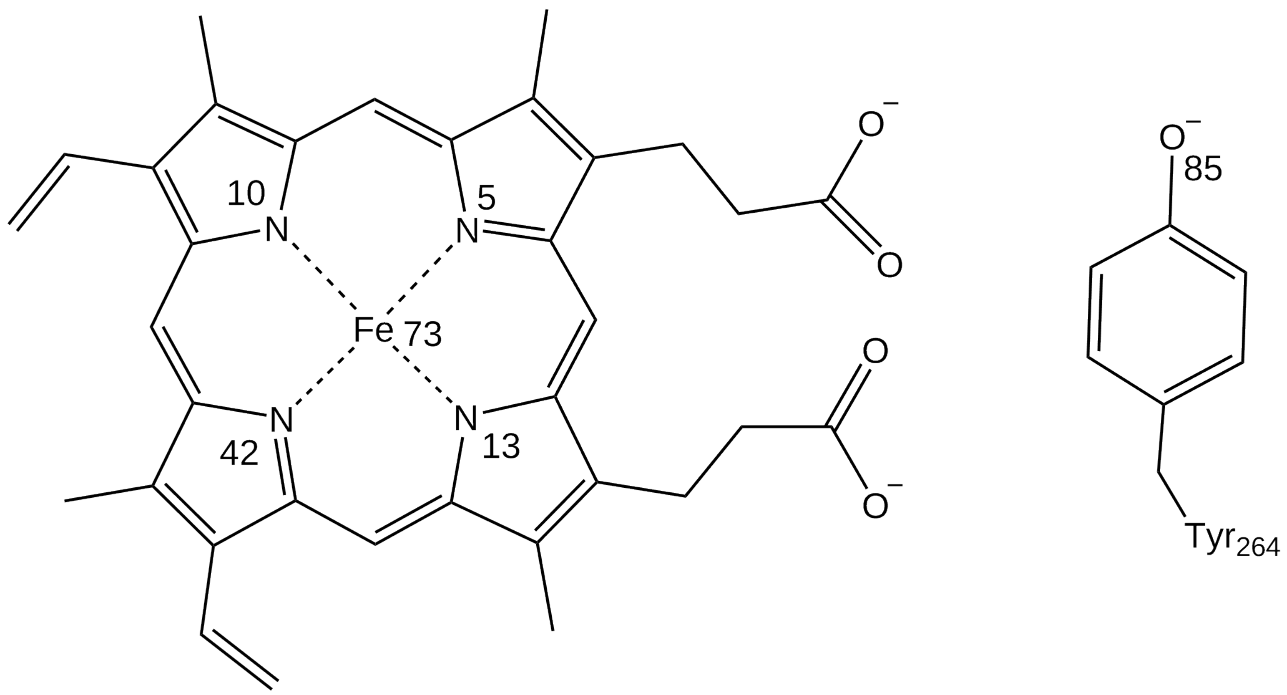


**Figure S30.** Atom numbering of the atoms considered in the bonded model between the Fe and the heme and Tyr264.

## References


1. Altschul, S.F., Madden, T.L., Schaäffer, A.A., Zhang. J., Zhang, Z., Miller. W. & Lipman, D.J. Gapped BLAST and PSI-BLAST: a new generation of protein database search programs. *Nucleic Acid Res.* **25**, 3389-3402 (1997).
2. Remmert, M., Biegert, A., Hauser, A. & Söding, J. HHblits: lightning-fast iterative protein sequence searching by HMM-HMM alignment. *Nat. Methods* **9,** 173-175 (2011).
3. 3.Arnold K, Bordoli L, Kopp J, Schwede T (2006) The SWISS-MODEL workspace: a web-based environment for protein structure homology modelling. Bioinformatics 22:195-201.
4. Benkert, P., Biasini, M. & Schwede, T. Toward the estimation of the absolute quality of individual protein structure models. *Bioinformatics* **27,** 343-350 (2011).
5. Biasini, M., Bienert. S., Waterhouse, A., Arnold, K., Studer, G., Schimdt, T., Kiefer, F., Gallo Cassarino, T., Bertoni, M., Bordoli, L. & Schwede, T. SWISS-MODEL: modelling protein tertiary and quaternary structure using evolutionary information. Nucleic Acid Res. **42,** W252-W258 (2014).
6. Williams, C.J., Headd, J.J., Moriarty, N.W., Prisant, M.G., Videau, L.L., Deis, L.N., Verma, V., Keedy, D.A., Hintze, B.J., Chen, V.B., Jain, S., Lewis, S.M., Arendall, W.B. 3$^{rd}$, Snoeyink, J., Adams. P.D., Lovell, S.C., Richardson, J.S. & Richardson, D.C. MolProbity: More and better reference data for improved all-atom structure validation. *Protein Sci.* **27,** 293-315 (2018).
7. Marana, S.R. Molecular basis of substrate specificity in family 1 glycoside hydrolases. *IUBMB Life* **58**, 63-73 (2006).
8. Kuusk, S. & Väljamäe, P. When substrate inhibits and inhibitor activates: implications of $\beta$-glucosidases. *Biotechnol. Biofuels* **10,** 7 (2017).
