# Supplementary material for: Heme-binding enables allosteric modulation in an ancient TIM-barrel glycosidase
Source: Nat Commun. 2021 Jan 15;12:380. doi: 10.1038/s41467-020-20630-1 (PMC7810902; doi:10.1038/s41467-020-20630-1)
Supplement: Supplementary file 4 — Description of additional supplementary files [file 41467_2020_20630_MOESM4_ESM.docx]

Description of Additional Supplementary files

Title: Supplementary Dataset

Description: Literature values of the optimum activity temperatures and host living temperatures for modern family 1 glycosidases. The relevant references are appended at the end of the table. See Methods in the main text for details on the performed literature search. Organismal living temperature is defined as the optimum growth temperature. For some organisms, optimum intervals or living temperature intervals are reported in the literature. Likewise, for some enzymes an interval of optimum activity temperature is provided in the literature. Organisms are classified as hyperthermophiles, extreme thermophiles, thermophiles, mesophiles and psycrophiles according to the descriptions reported in the literature. No classification is reported when such a description is not specifically provided in the literature, although most of the non-classified organisms are obviously mesophiles.
