# Supplementary material for: Heme-binding enables allosteric modulation in an ancient TIM-barrel glycosidase
Source: Nat Commun. 2021 Jan 15;12:380. doi: 10.1038/s41467-020-20630-1 (PMC7810902; doi:10.1038/s41467-020-20630-1)
Supplement: Supplementary file 5 — Reporting Summary [file 41467_2020_20630_MOESM5_ESM.pdf]

nature research

Corresponding author(s): Jose M. Sanchez-Ruiz

Last updated by author(s): Dec 8, 2020

Reporting Summary

Nature Research wishes to improve the reproducibility of the work that we publish. This form provides structure for consistency and transparency in reporting. For further information on Nature Research policies, see our Editorial Policies and the Editorial Policy Checklist.

Statistics

For all statistical analyses, confirm that the following items are present in the figure legend, table legend, main text, or Methods section.

n/a	Confirmed	
☐	☒	The exact sample size (*n*) for each experimental group/condition, given as a discrete number and unit of measurement
☒	☐	A statement on whether measurements were taken from distinct samples or whether the same sample was measured repeatedly
☒	☐	The statistical test(s) used AND whether they are one- or two-sided<br>*Only common tests should be described solely by name; describe more complex techniques in the Methods section.*
☒	☐	A description of all covariates tested
☒	☐	A description of any assumptions or corrections, such as tests of normality and adjustment for multiple comparisons
☐	☒	A full description of the statistical parameters including central tendency (e.g. means) or other basic estimates (e.g. regression coefficient) AND variation (e.g. standard deviation) or associated estimates of uncertainty (e.g. confidence intervals)
☒	☐	For null hypothesis testing, the test statistic (e.g. *F*, *t*, *r*) with confidence intervals, effect sizes, degrees of freedom and *P* value noted<br>*Give P values as exact values whenever suitable.*
☒	☐	For Bayesian analysis, information on the choice of priors and Markov chain Monte Carlo settings
☒	☐	For hierarchical and complex designs, identification of the appropriate level for tests and full reporting of outcomes
☒	☐	Estimates of effect sizes (e.g. Cohen's *d*, Pearson's *r*), indicating how they were calculated

Our web collection on statistics for biologists contains articles on many of the points above.

Software and code

Policy information about availability of computer code

Data collection	BLASTp (version 2.6.0) was used to retrieve GH1 homolog sequences from GenBank
Data analysis	T-coffee (version 9.03) was used to align protein sequences.<br>RAxML (version 8.2.11) was used to obtain initial, non-bootstrapped phylogenetic trees used as input for MrBayes.<br>MrBayes (version 3.2.6) was used to obtain final phylogenetic trees.<br>TastML (version 3.1) was used for the reconstruction of ancestral sequences.<br>CCP4 suite (version 7.0) was used to index and scale X-ray diffraction data.<br>PHENIX suite (version 1.14.3269) for 3D-structure refinement.<br>MODELLER (version 9.23) was used to reconstruct missing regions of protein structures for MD simulations.<br>PROPKA (version 3.1) was used to estimate pK values for protein ionisable residues.<br>AMBER19 was used to perform Molecular Dynamics simulations.<br>SWISS-MODEL was used to generate homology models of the ancestral glycosidase and to assess the quality of the models.<br>MolProbity (version 4.4) provided structural parameters used for assessing the quality of homology models.<br>PyMol (vesion 2.3.4) was used to generate images of protein structures.<br>LigPlot + (version 1.4.5) was used to generate 2D-interaction diagrams.<br>HeteroAnalysis (version 1.1.60) was used to analyze sedimentation equilibrium data.<br>SEDFIT (version 16.1c) was used to analyze sedimentation velocity data.<br>MO.Control (version 1.6) (NanoTemper Technologies GmbH) was used to analyze thermophoresis experiments.<br>SEDNTERP (version 20120111 Beta) was used for the interpretation of data from sedimentation velocity and sedimentation equilibrium experiments.

For manuscripts utilizing custom algorithms or software that are central to the research but not yet described in published literature, software must be made available to editors and reviewers. We strongly encourage code deposition in a community repository (e.g. GitHub). See the Nature Research guidelines for submitting code & software for further information.

April 2020

Data

Policy information about availability of data

All manuscripts must include a data availability statement. This statement should provide the following information, where applicable:

- Accession codes, unique identifiers, or web links for publicly available datasets
- A list of figures that have associated raw data
- A description of any restrictions on data availability

The data that supports the findings of this study are available within the article and its supplementary material. The crystallographic coordinates and the experimental structure factors have been deposited with the Protein Data Bank (https://www.rcsb.org) with ID 6Z1M and 6Z1H for the ancestral glycosidase with and without bound heme, respectively. Reconstructed ancestral sequences are available upon request and can also be found in these PDB files. Parameters used to describe the heme, input files, snapshots from our molecular dynamics simulations, and simulation trajectories (with water molecules and ions removed to save files size) are available for download from Zenodo (https://zenodo.org) at DOI: 10.5281/zenodo.3857791.

Field-specific reporting

Please select the one below that is the best fit for your research. If you are not sure, read the appropriate sections before making your selection.

☒ Life sciences ☐ Behavioural & social sciences ☐ Ecological, evolutionary & environmental sciences

For a reference copy of the document with all sections, see nature.com/documents/nr-reporting-summary-flat.pdf

Life sciences study design

All studies must disclose on these points even when the disclosure is negative.

Sample size	Not applicable. Our paper describes a protein engineering study in which biophysical methodologies are used to determine certain protein properties. It is not, for instance, a biomedical study or an animal behavior study, in which specific hypotheses are tested on the basis of statistical analyses of the results or observations on a number of individuals. Issues related to statistical hypothesis testing (such as sample size, randomization, replication or blinding) do not apply to our study. Of course, as it is customary in our field, crucial biophysical determinations are repeated a number of times (typically 3) to have an estimation of the error associated to the determined value. When doing so, we never excluded any data.
Data exclusions	No data have been excluded
Replication	Not applicable. Our paper describes a protein engineering study in which biophysical methodologies are used to determine certain protein properties. It is not, for instance, a biomedical study or an animal behavior study, in which specific hypotheses are tested on the basis of statistical analyses of the results or observations on a number of individuals. Issues related to statistical hypothesis testing (such as sample size, randomization, replication or blinding) do not apply to our study. Of course, as it is customary in our field, crucial biophysical determinations are repeated a number of times (typically 3) to have an estimation of the error associated to the determined value. When doing so, we never excluded any data.
Randomization	Not applicable. Our paper describes a protein engineering study in which biophysical methodologies are used to determine certain protein properties. It is not, for instance, a biomedical study or an animal behavior study, in which specific hypotheses are tested on the basis of statistical analyses of the results or observations on a number of individuals. Issues related to statistical hypothesis testing (such as sample size, randomization, replication or blinding) do not apply to our study. Of course, as it is customary in our field, crucial biophysical determinations are repeated a number of times (typically 3) to have an estimation of the error associated to the determined value. When doing so, we never excluded any data.
Blinding	Not applicable. Our paper describes a protein engineering study in which biophysical methodologies are used to determine certain protein properties. It is not, for instance, a biomedical study or an animal behavior study, in which specific hypotheses are tested on the basis of statistical analyses of the results or observations on a number of individuals. Issues related to statistical hypothesis testing (such as sample size, randomization, replication or blinding) do not apply to our study. Of course, as it is customary in our field, crucial biophysical determinations are repeated a number of times (typically 3) to have an estimation of the error associated to the determined value. When doing so, we never excluded any data.

Reporting for specific materials, systems and methods

We require information from authors about some types of materials, experimental systems and methods used in many studies. Here, indicate whether each material, system or method listed is relevant to your study. If you are not sure if a list item applies to your research, read the appropriate section before selecting a response.

Materials & experimental systems

n/a	Involved in the study	
☒	☐	Antibodies
☒	☐	Eukaryotic cell lines
☒	☐	Palaeontology and archaeology
☒	☐	Animals and other organisms
☒	☐	Human research participants
☒	☐	Clinical data
☒	☐	Dual use research of concern

Methods

n/a	Involved in the study	
☒	☐	ChIP-seq
☒	☐	Flow cytometry
☒	☐	MRI-based neuroimaging
